# The Molecular Cochaperone NbSGT1 May Function as an Endogenous Suppressor of RNA Silencing That Is Recruited by a Potyvirus in Infection of Plants

**DOI:** 10.1111/mpp.70221

**Published:** 2026-02-18

**Authors:** Wei Shi, Liwen Zhang, Na Li, Bei Gou, Li Qin, Wenping Qiu, Hongguang Cui, Hui Wang, Zhaoji Dai

**Affiliations:** ^1^ Key Laboratory of Green Prevention and Control of Tropical Plant Diseases and Pests (Ministry of Education), School of Tropical Agriculture and Forestry, Hainan University Haikou Hainan China; ^2^ School of Agriculture Science and Conservation Missouri State University Springfield Missouri USA; ^3^ Hainan Provincial Agricultural Machinery Appraisal and Extension Station Haikou Hainan China

**Keywords:** ESR, HC‐Pro, potyvirus, RNA silencing, SGT1, telosma mosaic virus

## Abstract

Recent evidence indicates that plant cells contain specific endogenous suppressors of RNA silencing (ESRs) that plant viruses can exploit to counteract host defences. However, the underlying mechanisms are not yet fully understood. The helper component‐proteinase (HC‐Pro) of potyviruses is known to suppress RNA silencing and facilitate viral infection. Here, we used affinity purification followed by mass spectrometry to identify potential host proteins that interact with HC‐Pro during telosma mosaic virus (TelMV, genus *Potyvirus*) infection in plants. We found that the molecular co‐chaperone SGT1 (suppressor of the G2 allele of Skp1) interacts with HC‐Pro via its SGS domain. Virus‐induced gene silencing and RNAi‐mediated knockdown of *NbSGT1* resulted in decreased viral accumulation in *Nicotiana benthamiana* plants. Conversely, transient overexpression of *NbSGT1* promoted TelMV multiplication. Through alanine‐scanning mutagenesis, we identified three residues (K217, I227 and E332) in HC‐Pro that are essential for its interaction with NbSGT1. Mutant viruses carrying these mutations exhibited reduced viral accumulation without affecting the RNA suppression of silencing (RSS) activity of HC‐Pro. NbSGT1 enhanced the RSS activity of HC‐Pro and increased the expression of foreign genes at both the protein and mRNA levels. Additionally, we demonstrated, through an *Agrobacterium* infiltratrion assay, that NbSGT1 may act as an ESR, inhibiting local but not systemic RNA silencing in plants. Finally, NbSGT1 significantly downregulated the expression of *AGOs*, *DCLs*, *RDRs* and *SGS3*, key genes of the RNA silencing pathway. Collectively, our findings provide evidence that the molecular co‐chaperone SGT1 may act as a host ESR and is recruited by a potyvirus to facilitate viral infection.

## Graphical abstract

The molecular co‐chaperone NbSGT1 interacts with potyviral HC‐Pro and promotes viral infection in plants. Notably, (1) NbSGT1 enhances the RSS activity of HC‐Pro and (2) NbSGT1 acts as an endogenous suppressor of RNA silencing (ESR), suppressing RNA silencing in plants.

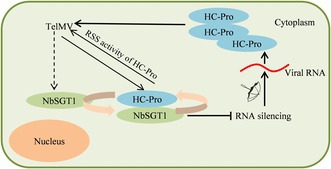

## 1 Introduction

RNA silencing is a conserved, sequence‐specific machinery that regulates gene expression in eukaryotes through transcriptional gene silencing (TGS) and post‐transcriptional gene silencing (PTGS) (Pumplin and Voinnet 2013; Baulcombe 2022). Plant viruses are obligate intracellular parasites that can cause devastating diseases for plants (Dai and Wang 2022; Wang 2015). To combat viral infection, plants use RNA silencing as a defence mechanism by targeting viral RNA for degradation. The classic core pathway of RNA silencing in plants involves two main steps. First, an RNA‐dependent RNA polymerase (RDR) synthesises double‐stranded RNA (dsRNA) from a single‐stranded precursor. Plants use Dicer‐like endoribonucleases (DCLs) to cleave this dsRNA into small interfering RNAs (siRNAs). Then, these siRNAs are incorporated into the Argonaute (AGO) nuclease‐containing RNA‐induced silencing complex (RISC) to cleave homologous target RNAs (Wang et al. 2021; Baulcombe 2022; Li et al. 2025). To counteract the host defence by RNA silencing, viruses have evolved to encode proteins that function as suppressors of RNA silencing, commonly known as viral suppressors of RNA silencing (VSRs) or RNA silencing suppressors (RSSs) (Li and Ding 2001; Csorba et al. 2015; Jin et al. 2021). Some of the most commonly studied VSRs include the cucumoviral 2b protein, the tombusviral p19, and the potyviral HC‐Pro protein.

In addition to the numerous VSRs, endogenous suppressors of RNA silencing (ESRs) have been reported in plants. The first reported ESR in plants was a *Nicotiana tabacum* calmodulin‐related protein, known as rgs‐CaM (Anandalakshmi et al. 2000). Since then, several other plant ESRs have been reported, including reg‐CaM from *Nicotiana benthamiana* (Li et al. 2014), NgRBP from *Nicotiana glutinosa* (Huang et al. 2019), RLI2 (ABCE2), FIERY1, XRN2, XRN3, XRN4 and SKI3 from *Arabidopsis thaliana* , as well as StLTP6 from *Solanum tuberosum* (Gazzani et al. 2004; Sarmiento et al. 2006; Gy et al. 2007; Yu et al. 2015; Shang et al. 2022). Recent findings have demonstrated that ESRs can be exploited by plant viruses, including geminiviruses, potato virus X and potato virus S (Li et al. 2014, 2017; Huang et al. 2019), to couteract host defences. However, the underlying mechanisms of this process are not fully understood.

Passion fruit ( *Passiflora edulis* ) is a perennial vine‐like fruit tree that primarily grows in tropical and subtropical regions (Wang et al. 2024). However, viral diseases are a major constraint for passion fruit production. Telosma mosaic virus (TelMV, *Poytvirus*) is the most common viral pathogen infecting passion fruit worldwide. Over the years, TelMV has also been reported to infect several other plant species, including *N. benthamiana*, *Pogostemon cablin* , *Chenopodium quinoa* , *Chenopodium amaranticolor* , *Phaseolus vulgaris* and *Senna alata* (Wang et al. 2025). However, there was still a lack of studies on TelMV–host interactions. The helper component‐proteinase (HC‐Pro) of plant potyviruses, the first reported VSR (Anandalakshmi et al. 1998; Kasschau and Carrington 1998), is a multifunctional protein involved in aphid transmission, protease activity, virus replication, virus movement and gene silencing suppression (Valli et al. 2018; Wang et al. 2024). Our recent study has demonstrated that the HC‐Pro protein encoded by TelMV acts as an RSS (Wang et al. 2024).

Suppressor of G2 allele of skp1 (SGT1), known as a molecular co‐chaperone, is highly conserved in eukaryotes and functions in various aspects of plant biology, including development and immune responses (Zhang et al. 2024; Meldau et al. 2011). SGT1 was first reported to be involved in *R* gene‐triggered disease resistance (Azevedo et al. 2002; Austin et al. 2002). In the same year, SGT1 was first reported to participate in tobacco mosaic virus (TMV) infection in plants (Liu, Schiff, Marathe, and Dinesh‐Kumar 2002; Liu, Schiff, Serino, et al. 2002). Since then, SGT1 has been documented to be involved in the infection processes of various plant viruses, including potato virus X (PVX) (Ye et al. 2012), tomato yellow leaf curl virus (TYLCV) (Moshe et al. 2016), tomato spotted wilt virus (TSWV) (Qian et al. 2018; Chen et al. 2021) and tomato chlorosis virus (ToCV) (Ontiveros et al. 2023). However, the underpinning mechanisms by which SGT1 regulates viral infection are not yet fully understood. Additionally, although potyviruses represent the largest group of known plant RNA viruses, it is still not clear whether SGT1 participates in the potyviral infection.

This study aims to identify the host proteins associated with HC‐Pro in the context of TelMV infection and to investigate the role of HC‐Pro‐interacting protein, NbSGT1, during TeMV infection. We found that NbSGT1 interacts with HC‐Pro, enhances its RSS activity and promotes TelMV infection in plants. NbSGT1 also promotes foreign gene expression at both the protein and mRNA levels. Furthermore, NbSGT1 functions as ESR, inhibiting local RNA silencing in *N. benthamiana* plants. Lastly, it downregulates the expression of key genes in the RNA silencing pathway. Collectively, our data provide evidence that NbSGT1 interferes with RNA silencing but promotes potyvirus infection in plants.

## 2 Results

### 2.1 Construction of TelMV Infectious Clone With Twin‐Strep‐Tagged HC‐Pro

**FIGURE 1:**
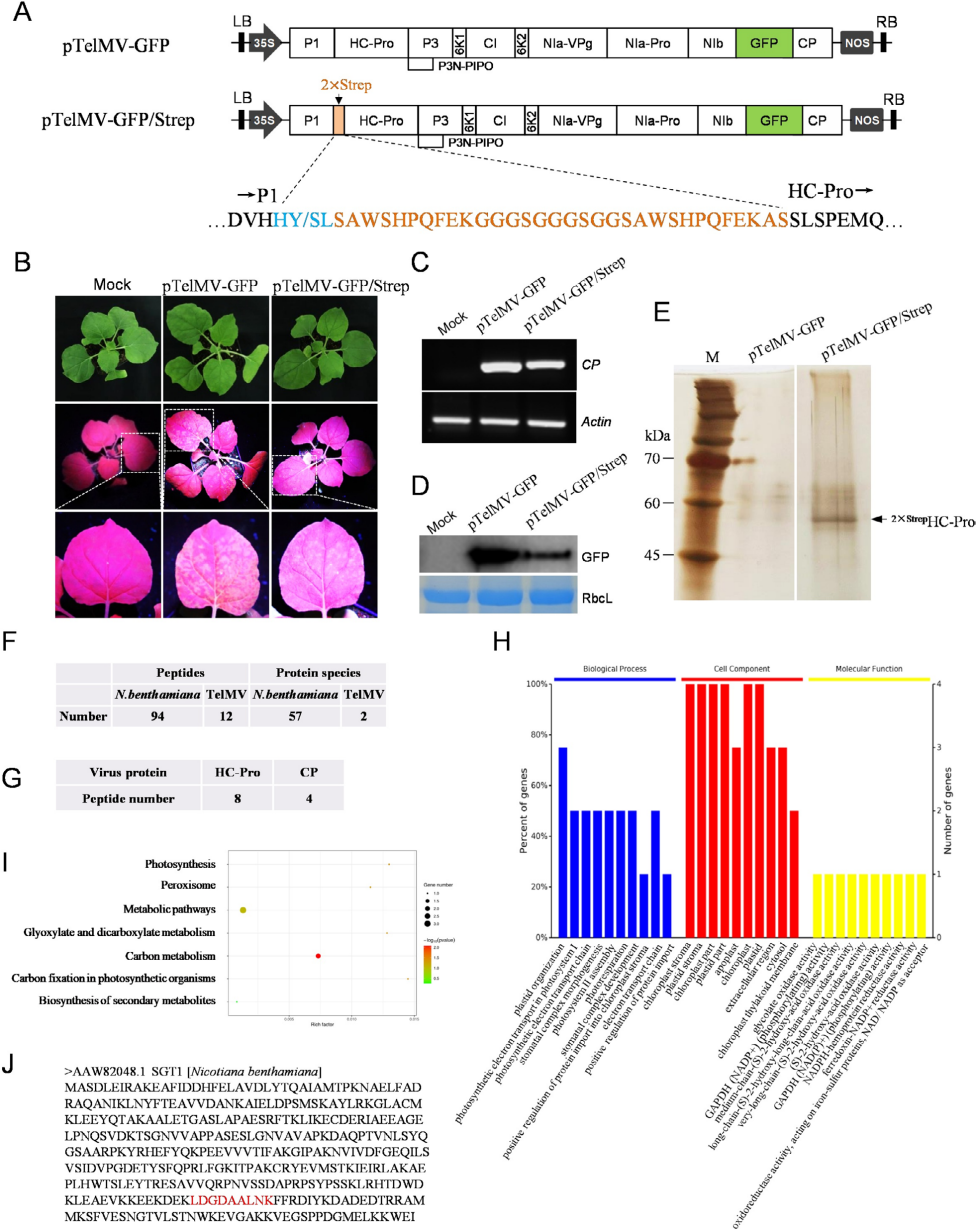
Identification of HC‐Pro‐interacting proteins in *Nicotiana benthamiana* plants. (A) Schematic representation of recombinant telosma mosaic virus (TelMV) infectious clones. Upper panel: GFP‐tagged TelMV infectious clone, pTelMV‐GFP; Lower panel: Twin‐Strep‐tagged TelMV infectious clones, pTelMV‐GFP/Strep. The orange rectangle represents the Twin‐Strep‐tag, the green rectangle represents the GFP cistron, blue represents the P1/HC‐Pro cleavage site. (B) *N. benthamiana* plants inoculated by buffer (Mock), positive control (pTelMV‐GFP) or pTelMV‐GFP/Strep, under UV light at 10 days post‐infiltration (dpi). (C) Reverse transcription‐PCR analyses of *CP* transcripts in the systemically infected leaf of *N. benthamiana* shown in (B). The *N. benthamiana Actin* gene serves as the internal control. (D) Western blot analyses of GFP in the systemically infected leaf of *N. benthamiana* shown in (B). The Coomassie Brilliant Blue (CBB)‐stained RuBisCO large subunit (RbcL) serves as a loading control. (E) Silver staining of SDS‐PAGE analysis for pTelMV‐GFP or pTelMV‐GFP/Strep after elution from Strep‐Tactin MacroPrep resin. The arrow indicates the estimated size of the 2 × Strep‐HC‐Pro product. (F) LC–MS/MS identification of co‐purified products associated with 2 × Strep‐HC‐Pro. (G) Summary of viral proteins co‐purified with 2 × Strep‐HC‐Pro in *N. benthamiana* plants. (H) GO enrichment analysis. The biological process, cellular component and molecular function of GO enrichment analysis are shown as blue, red and yellow bars, respectively. (I) KEGG pathway analysis. Bubble colour and size distribution represent −log*p* values and the number of genes involved in each signalling pathway, respectively. (J) Amino acid sequence of NbSGT1 (GenBank: AAW82048.1). The peptide sequence of NbSGT1 identified by LC–MS/MS is marked in red.

The classic Strep tag is a short peptide consisting of eight amino acids (WSHPQFEK) and is widely used for affinity purification of recombinant proteins. An advanced version, the Twin‐Strep‐tag (WSHPQFEKGGGSGGGSGGSAWSHPQFEK), offers significantly higher binding affinity and additional advantages (Schmidt et al. 2013; Saribas et al. 2020). Hence, we decided to construct a TelMV infectious clone bearing Two‐Strep‐tagged HC‐Pro, which would enable us to pull down proteins associated with HC‐Pro in the TelMV‐infected plant cells. It is well known that the potyviral genome is primarily restricted to the P1/HC‐Pro and NIb/CP junctions for the insertion of foreign fragments (Xie et al. 2021; Wang et al. 2025). We decided to insert the Twin‐Strep tag at the N‐terminal of the HC‐Pro cistron in the previously reported GFP‐tagged TelMV infectious clone (pTelMV‐GFP) created by our group (Gou et al. 2023) (Figure 1A, upper panel). This modification allows for convenient detection and monitoring of viral infection. The resulting recombinant virus was named pTelMV‐GFP/Strep (Figure 1A, lower panel).

Next, pTelMV‐GFP/Strep and the control vector pTelMV‐GFP were electroporated into *Agrobacterium tumefaciens* GV3101 and subsequently delivered into fully expanded leaves of 3‐ to 4‐week‐old *N. benthamiana* plants by agroinfiltration. As expected, green fluorescence was clearly observable under UV illumination in the upper uninoculated leaves of pTelMV‐GFP‐inoculated plants at 10 days post‐infiltration (dpi). Similarly, the upper leaves of the pTelMV‐GFP/Strep‐infiltrated plants displayed distinct green fluorescence at 10 dpi (Figure 1B), indicating that pTelMV‐GFP/Strep had successfully induced systemic viral infection in plants. Additionally, the systemic infection of pTelMV‐GFP/Strep was validated by reverse transcription‐quantitative PCR (RT‐qPCR) and western blot analyses, which detected viral RNA and GFP protein, respectively (Figure 1C,D). Together, these results demonstrated that virus with the added Twin‐Strep tag on the N‐terminus of HC‐Pro established successful systemic infection in *N. benthamiana* plants.

### 2.2 Identification of Viral and Host Proteins Associated With HC‐Pro During Viral Infection in Plants

To pull down proteins associated with HC‐Pro in the context of viral infection, we inoculated 3‐ to 4‐week‐old *N. benthamiana* plants using the above‐described Strep‐tagged TelMV infectious clone, pTelMV‐GFP/Strep. At 15 dpi, the systemic leaves exhibiting green fluorescence under UV illumination were harvested, pooled and subjected to a pulldown assay with Strep‐Tactin resin. Protein samples were washed and then eluted with biotin, followed by resolution using SDS‐PAGE, and finally visualised through silver staining. As shown in Figure 1E, the pTelMV‐GFP/Strep produced a distinct protein complex with a size of around 55 kDa, corresponding to the estimated size of 2 × Strep‐HC‐Pro. Finally, the purified protein complexes were subjected to protein identification by liquid chromatography–tandem mass spectrometry (LC–MS/MS).

The LC–MS/MS identified two viral proteins, HC‐Pro and CP (Figure 1F,G). Additionally, 57 host proteins were identified (Table 1). The gene ontology (GO) functional analysis categorised these host proteins into three areas: biological process, cell component and molecular function (Figure 1H). KEGG pathway analysis showed that the most abundant host proteins were associated with metabolic pathways and carbon metabolism (Figure 1I). Among the identified host proteins, we were particularly interested in proteins related to plant immunity. Notable proteins involved in plant immunity included GADPH CysP6, RbCS and SGT1 (Table 1, Figure 1J). Given SGT1's significant role in viral infections and the lack of reports on its role in potyvirus infection, we decided to focus our investigation on the potential role of SGT1 in TelMV infection.

**TABLE 1:** List of host proteins that are uniquely identified from co‐purified products with 2 × Strep‐HC‐Pro by LC–MS/MS.

No.	Uniprot	Protein description	Score
1	A0A481NUV9	Ferredoxin–NADP(+) reductase	323.31
2	J7ES67	Catechol oxidase	323.31
3	A0A0A8IBT8	Glyceraldehyde‐3‐phosphate dehydrogenase	311.08
4	A0A6H0CCF4	ATP synthase subunit α	98.19
5	A0A1S5WM37	Papain‐like cysteine proteinase 6	41.69
6	W6JLY3	Nuclear pore complex protein Nup136b	39.25
7	A0A8K1ZRH5	UDP‐glycosyltransferase	37.58
8	K0IBB4, C6FFS2	Catalase	29.66
9	E0X585	Glycine dehydrogenase (aminomethyl‐transferring)	27.85
10	H9C954	Actin	27.32
11	A0A286RNF7, A4D0J9, A4D0K0	Carbonic anhydrase	26.55
12	A0A1W5XFS7, C9DFB9, D4P5A4	HSP70	19.82
13	A0A0F7JIC2, A0A0F7JJ49, A0A0F7JLU6	Glyceraldehyde‐3‐phosphate dehydrogenase	17.36
14	W6KDG8	Green fluorescent protein	15.19
15	A0A0F7R5Z5, B6EBE6, I3QHX5	Adenosylhomocysteinase	14.16
16	Q6XX19	Translation elongation factor 1 α	13.78
17	A0A0S4IJL0	Ribulose bisphosphate carboxylase small subunit, chloroplastic	13.28
18	R9W4N2	Thioredoxin‐dependent peroxiredoxin	12.70
19	E5LLE7	Phosphoglycerate kinase	12.66
20	A0A6M4RTX4	DNA‐binding bromodomain‐containing protein	11.60
21	W6JJB4, W6JLY5	Nuclear pore complex protein TPRb; Nuclear pore complex protein TPRa	11.53
22	A0A0G2RIY7	Prolyl endopeptidase	10.81
23	A0A248QJL3, A0A248QFH8, A0A248QEL2	S‐adenosylmethionine synthase	10.08
24	Q5EFR5, I0B7K0, I0B7J9	16 kDa subunit of oxygen evolving system of photosystem II	9.00
25	E1AXT8	(S)‐2‐hydroxy‐acid oxidase	7.90
26	Q5EEQ1	Photosystem I reaction centre subunit X PSI‐K	7.74
27	A0A387K109, A0A387K3P5, A0A387K371	GTP‐binding nuclear protein	7.70
28	B2Z6N3	Neuroblastoma‐amplified protein	6.63
29	A0A0E3JCP4	Developmentally‐regulated plasma membrane polypeptide	6.57
30	U5YSN4, I0B7J8, I0B7J7	23 kDa subunit of oxygen evolving system of photosystem II	6.49
31	Q84KK8	Respiratory burst oxidase homologue	6.23
32	Q2LAH1, Q2LAH0	Chloroplast photosystem II 22 kDa component	6.21
33	A0A068GNX3	Domains rearranged methyltransferase 1	6.20
34	Q93YF7	Calcium‐dependent protein kinase 2	6.14
35	Q5EEQ0	Zinc finger protein	6.13
36	F8WQS3	Ascorbate peroxidase	6.08
37	G9BF72	MPB2C‐like protein	6.06
38	A0A224AKY6, Q84UV7, Q5EEY5	Suppressor of G2 allele of SKP1	6.01
39	A0A813M2E5	β‐hexosaminidase	5.96
40	G5DBJ0	Protein CHAPERONE‐LIKE PROTEIN OF POR1, chloroplastic	5.92
41	A0A8K1ZRH3	Glycosyltransferase	5.92
42	A7M8K5	Ran GTPase activating protein	5.92
43	A0A2I8B6Q8	Membrane‐localised LRR receptor‐like protein	5.92
44	A0A1V1H612	Respiratory burst oxidase homologue protein B	5.88
45	W6JM01	Nuclear pore complex protein Nup155b	5.87
46	E1AWW8	Nuclear localised protein 1	5.87
47	A0A0H5AZC9, A0A0H5AWF5	PDR‐type ACB transporter	5.83
48	A0A5J6DCT7	NLR‐required for cell death 4	5.82
49	U3MY90	Proteinase inhibitor	5.81
50	A0A3S7JLZ4	APETALA1	5.81
51	J7MBJ6	Protein kinase	5.7448
52	W6JLE5	Nuclear pore complex protein Nup136a	5.74
53	D5JXY5	Calcium‐transporting ATPase	5.72
54	A0A0P0INT0	ATP‐dependent RNA helicase	5.72
55	B0CN62	Myosin VIII‐1	5.71
56	A0A387K1P8, A0A387K491	Ran binding protein RanBP1‐1a, Ran binding protein RanBP1‐1b	5.70
57	A0A0K1U1W6	Clade XII lectin receptor kinase	5.68

### 2.3 NbSGT1 Interacts With HC‐Pro via Its SGS Domain

**FIGURE 2:**
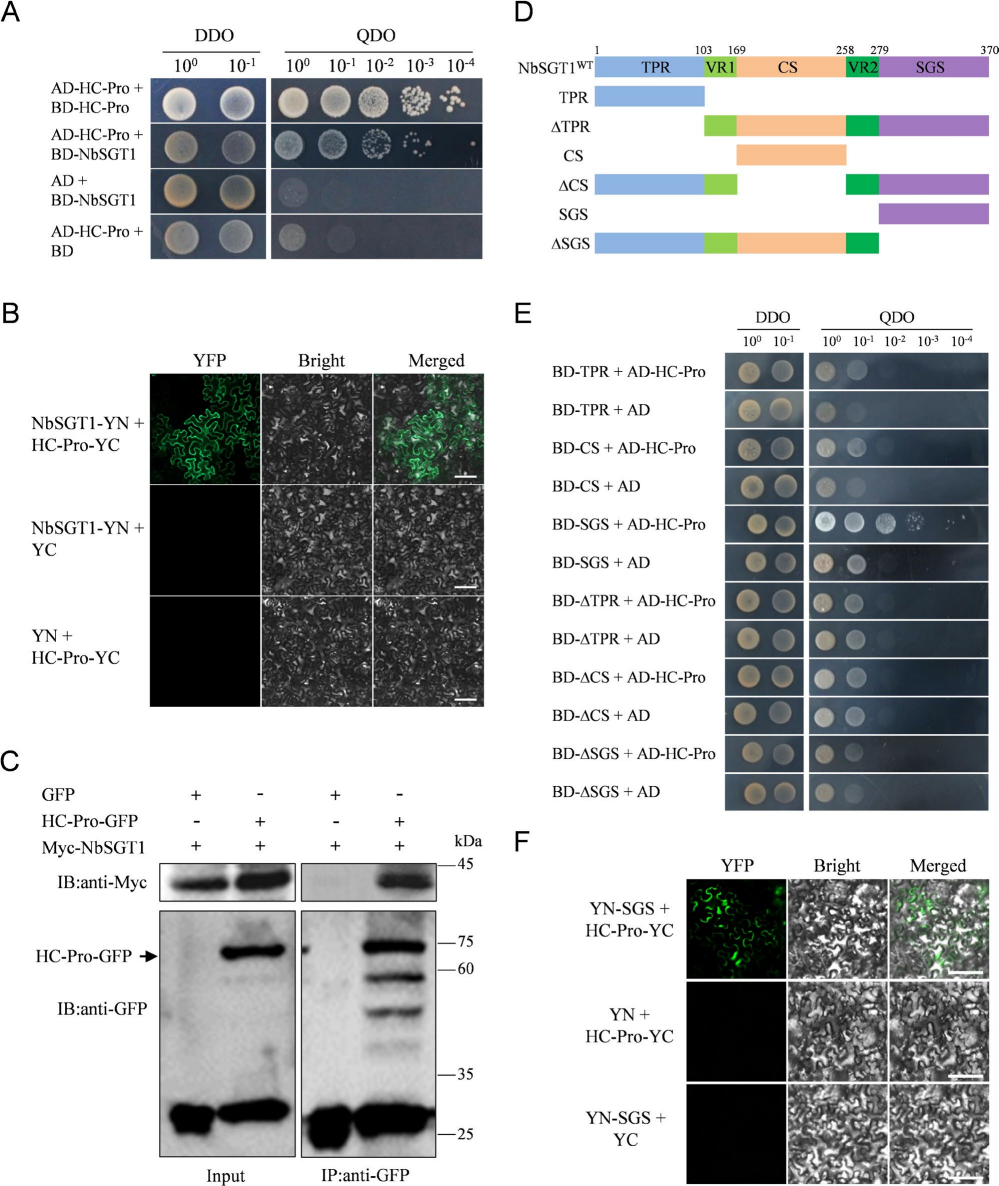
Telosma mosaic virus (TelMV) HC‐Pro interacts with NbSGT1 through the SGS domain. (A) HC‐Pro interacts with NbSGT1 in yeast. DDO: Double dropout medium lacking tryptophan and leucine; QDO: quadruple dropout medium lacking tryptophan, leucine, histidine and adenine. (B) Bimolecular fluorescence complementation (BiFC) analysis of HC‐Pro‐NbSGT1 interaction in *Nicotiana benthamiana* epidermal cells under a confocal microscope at 72 h post‐inoculation (hpi). Bars, 100 μm. (C) Co‐immunoprecipitation of HC‐Pro with NbSGT1. Protein samples before (Input) and after (Co‐IP) immunopurification were analysed by immunoblotting using anti‐GFP or anti‐Myc antibody. (D) Schematic representation of wild‐type (WT) and deletion mutants of *NbSGT1*. TPR: a tetratricopeptide repeat domain; CS: present in metazoan CHORD and SGT1 proteins; VR1 and VR2: two variable regions; SGS: SGT1‐specific motif. (E) The SGS domain interacts with HC‐Pro in yeast. (F) BiFC analysis of SGS–HC‐Pro interaction in *N. benthamiana* epidermal cells under a fluorescent microscope at 72 hpi. Bars, 100 μm.

To rule out the possibility of a nonspecific interaction between HC‐Pro and NbSGT1 identified through Strep‐tag affinity purification, we first investigated whether NbSGT1 could interact with HC‐Pro in yeast using the yeast two‐hybrid (Y2H) system. HC‐Pros are proteins well‐known to be capable of self‐interaction, thus serving as the positive control (Valli et al. 2018). As shown in Figure 2A, only the positive control and the co‐transformants of NbSGT1 and HC‐Pro were able to grow on quadruple dropout (QDO) medium, suggesting NbSGT1 can interact with HC‐Pro in yeast. In contrast, co‐transformation of BD‐NbSGT1 and AD empty vector or AD‐HC‐Pro and BD empty vector did not result in growth on the QDO medium.

To further validate the interaction between NbSGT1 and HC‐Pro in plant cells, we conducted a bimolecular fluorescence complementation (BiFC) assay in *N. benthamiana* leaves. NbSGT1 and HC‐Pro were fused to the N‐ or C‐terminal YFP fragment (YN or YC), respectively. Co‐expression of NbSGT1‐YN and HC‐Pro‐YC resulted in strong green fluorescence at 72 h post‐inoculation (hpi) under a confocal microscope (Figure 2B), suggesting NbSGT1 interacts with HC‐Pro in *N. benthamiana* leaf cells. Coexpression of the empty vector YN and HC‐Pro‐YC or NbSGT1‐YN and the empty vector YC yielded no fluorescence at 72 hpi (Figure 2B). Lastly, we conducted a co‐immunoprecipitation (Co‐IP) experiment to verify NbSGT1–HC‐Pro interaction in plants. To this end, GFP and Myc tags were fused to HC‐Pro and NbSGT1, respectively. The resulting recombinant proteins were then expressed in *N. benthamiana* leaves through agroinfiltration. Protein extracts were prepared from infiltrated leaves and used for the Co‐IP experiment with GFP‐Trap agarose. Immunoblot analysis revealed that NbSGT1 could be precipitated by HC‐Pro‐GFP, but not by free GFP (Figure 2C), suggesting that NbSGT1 interacts with HC‐Pro in *N. benthamiana* plants.

The coding sequence of NbSGT1 contains 1113 nucleotides encoding a protein with 370 amino acids and a molecular mass of approximately 41 kDa. Amino acid sequence alignment using SGT1 sequences from various species showed that, like other plant SGT1 proteins, NbSGT1 also contains five typical domains (a tetratricopeptide repeat domain [TPR], the CS motif [present in metazoan CHORD and SGT1 proteins], two variable regions [VR1 and VR2] and the SGS motif [SGT1‐specific motif]) (Figure 2D; Figure S1). Additionally, the phylogenetic tree revealed that NbSGT1 is closely related to the SGT1s from *Solanum lycopersicum* (Figure S2). To identify the domain of NbSGT1 responsible for its interaction with HC‐Pro, five mutant plasmids were constructed based on the predicted conserved domains (Figure 2D). The Y2H assays showed that the SGS domain, but not the TPR or CS domains, interacted with HC‐Pro (Figure 2E). A BiFC analysis was carried out to validate the interaction between the SGS domain and HC‐Pro in plants. The BiFC analysis revealed that co‐expression of SGS fused with the N‐terminal YFP (YN‐SGS) and HC‐Pro fused with the C‐terminal YFP (HC‐Pro‐YC) yielded strong green fluorescence under a fluorescent microscope (Figure 2F). These results indicate that the SGS domain of NbSGT1 is the key region responsible for its interaction with HC‐Pro.

### 2.4 Silencing *NbSGT1* Greatly Reduces Both the Local and Systemic Infections of TelMV in Plants

To investigate the biological significance of *NbSGT1* in TelMV infection, we employed the classic tobacco rattle virus (TRV)‐based virus‐induced gene silencing (VIGS) approach and tested the effect of silencing *NbSGT1* on TelMV infection in *N. benthamiana* plants. A 546 bp‐fragment of *NbSGT1* was cloned into the TRV vector, and the resulting vector (TRV:NbSGT1) was subsequently inoculated into the leaves of *N. benthamiana* plants. The silencing efficiency was assessed by RT‐qPCR at various time points (13, 17 and 21 dpi). The RT‐qPCR results revealed the successful silencing of *NbSGT1*, with an efficiency of 63%, 78% and 60% at the three time points (Figure S3, right panel). Interestingly, unlike the Mock and TRV:GUS‐inoculated plants, plants inoculated with TRV:NbSGT1 exhibited atypical growth phenotypes, including more branched and shorter shoots, as well as curly and mosaic leaves (Figure S3). This suggests that the silencing of the endogenous *NbSGT1* led to abnormal growth of *N. benthamiana* plants. These findings are consistent with previous observations regarding growth phenotype in NbSGT1‐silenced *N. benthamiana* plants (Peart et al. 2002; Yu et al. 2019).

**FIGURE 3:**
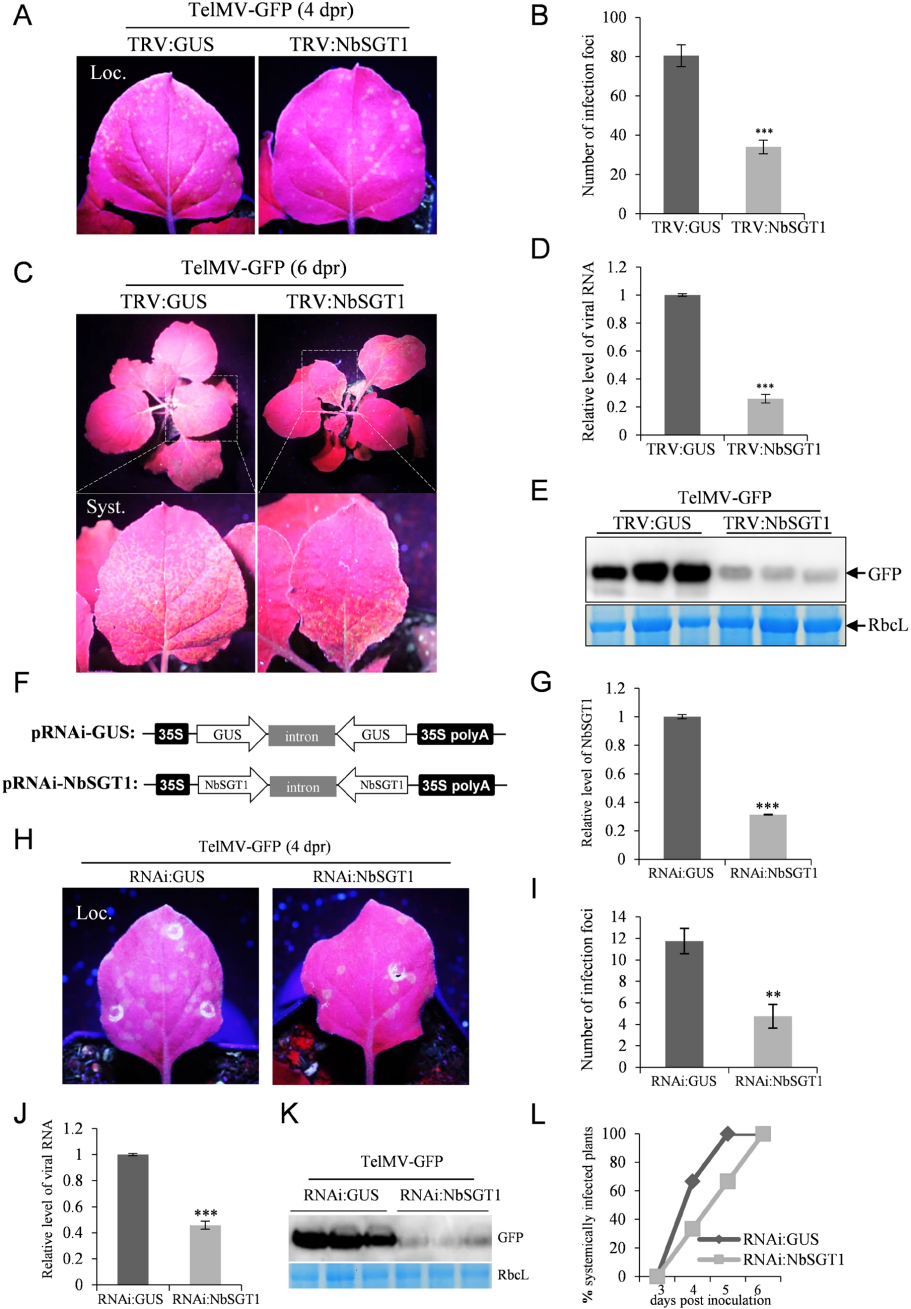
Virus‐induced gene silencing and RNAi‐mediated knockdown of *NbSGT1* resulted in decreased accumulation of telosma mosaic virus (TelMV) in *Nicotiana benthamiana* plants. (A) Silencing of *NbSGT1* through virus‐induced gene silencing (VIGS) inhibited the local infection of TelMV‐GFP in plants at 4 days post‐rub‐inoculation (dpr). Regular, regular light. UV, ultraviolet light. Loc., local leaf. (B) The number of infection foci on the local leaves of TRV‐GUS or TRV‐NbSGT1‐inoculated plants by TelMV‐GFP infection at 4 dpr. Green fluorescent foci were counted under UV light. (C) Silencing of *NbSGT1* through VIGS delayed the systemic infection of TelMV‐GFP in plants at 6 dpr. Syst., systemic leaf. (D) Reverse transcription‐quantitative PCR (RT‐qPCR) analyses of viral RNA levels sampled from the systemic leaves of TRV‐GUS or TRV‐NbSGT1‐inoculated plants upon TelMV infection at 6 dpr. (E) Western blot analysis of GFP in the systemic leaf of *NbSGT1*‐silenced plants infected by TelMV‐GFP at 6 dpr. (F) Schematic illustration of RNAi constructs for transient gene silencing in plants. The RNAi constructs contain both sense and antisense *GUS* or *NbSGT1* sequences separated by an intron. (G) RT–qPCR analysis for detecting *NbSGT1* transcripts in RNAi construct‐inoculated plants at 72 days post‐inoculation. (H) Comparison of the fluorescent foci induced by TelMV‐GFP infection between *NbSGT1*‐silenced and non‐silenced leaves under UV light at 4 dpr. (I) Number of infection foci on the local leaves of RNAi‐GUS or RNAi‐NbSGT1‐inoculated plants by TelMV‐GFP infection at 4 dpr. Green fluorescent foci were counted under UV light at 4 dpr. (J) RT‐qPCR analyses of viral RNA levels sampled from the local leaves of RNAi‐GUS or RNAi‐NbSGT1‐inoculated plants upon TelMV infection at 4 dpr. (K) Western blot analysis of GFP in the local leaf of RNAi‐NbSGT1‐inoculated plants upon TelMV‐GFP infection at 4 dpr. (L) Plot shows the percentage of plants that exhibited systemic TelMV‐GFP infection between 3 and 6 dpi. The Coomassie Brilliant Blue‐stained RbcL serves as a loading control. The transcript levels were normalised against the internal control, *NbActin*. Error bars denote standard error and Student's *t* test was applied to determine statistical significance (**p < 0.01; ***p < 0.001). All experiments were repeated with similar results.

Next, the effect of silencing of *NbSGT1* on TelMV infection was investigated. To this end, we rub‐inoculated the *NbSGT1*‐silenced plants with TelMV‐GFP and tested the capability of viral infection by visual observations and molecular detections. In the local leaves, rub‐inoculation of TelMV‐GFP resulted in fluorescent infection foci upon UV illumination (Figure 3A). Statistical analysis revealed a significant decrease in number of infection foci in the TRV:NbSGT1‐inoculated plants compared to the control plants inoculated with TRV:GUS at 4 days post‐rub‐inoculation (dpr) (Figure 3B). We monitored the viral infection in TRV:NbSGT1‐inoculated plants and found greatly reduced green fluorescence in the upper non‐inoculated leaves compared to the control plants at 6 dpr (Figure 3C). The systemic leaves were sampled and subjected to RT‐qPCR and western blot analyses for the detection of viral RNA and GFP protein, respectively. The results revealed a large reduction of both viral RNA and GFP protein in the systemic leaves of *NbSGT1*‐silenced plants compared to the control plants (Figure 3D,E). The presence of TRV:GUS as well as TRV:NbSGT1 in the respective plant tissues were also confirmed by RT‐PCR using TRV‐specific primers (Figure S4). Together, these results demonstrated that silencing *NbSGT1* greatly hampered both the local and systemic infection of TelMV in *N. benthamiana* plants.

### 2.5 RNAi‐Induced Transient Gene Silencing of *NbSGT1* Reduces Local Infection and Delays the Systemic Infection of TelMV in Plants

Intron‐containing constructs encoding self‐complementary hairpin RNA (ihpRNA) have been broadly used for RNAi‐induced transient gene silencing in various plant species (Hoffmann et al. 2006). To further validate the silencing effect of NbSGT1 on TelMV infection, we performed transient ihpRNAi‐induced gene silencing in *N. benthamiana* plants. The vector pRNAi‐NbSGT1 (Figure 3F) was generated by inserting a 546‐bp fragment of the *NbSGT1* gene in the sense and antisense orientation interrupted by an intron in the hpRNAi construct p2300s‐intron vector (Hu et al. 2025). An *Agrobacterium* suspension harbouring the pRNAi‐NbSGT1 construct was infiltrated into *N. benthamiana* plants. At 72 hpi, RT‐qPCR analysis revealed that endogenous *NbSGT1* was silenced by 69% in the local leaves (Figure 3G).

Next, we rub‐inoculated the pRNAi‐NbSGT1‐infiltrated plants at 36 hpi using sap prepared from TelMV‐GFP‐infected *N. benthamiana* plants. At 4 dpr, fewer fluorescent foci could be observed in pRNAi‐NbSGT1‐inoculated leaves compared to the control leaves infiltrated with pRNAi‐GUS (Figure 3H,I), suggesting silencing *NbSGT1* reduced TelMV‐GFP infection in plants. Additionally, RT‐qPCR and western blot analyses also confirmed the lower accumulation levels of viral RNA and GFP protein in the *NbSGT1*‐silenced leaves (Figure 3J,K). Furthermore, we monitored the viral systemic infection by observing green fluorescence in the upper non‐inoculated leaves in a time‐course manner, and found that transient silencing of *NbSGT1* delayed the systemic infection of TelMV‐GFP (Figure 3L). Taken together, the RNAi‐induced gene silencing of *NbSGT1* weakened the local infection and delayed the systemic infection of TelMV in plants.

### 2.6 Transient Overexpression of *NbSGT1* Promotes TelMV Infection in Plants

**FIGURE 4:**
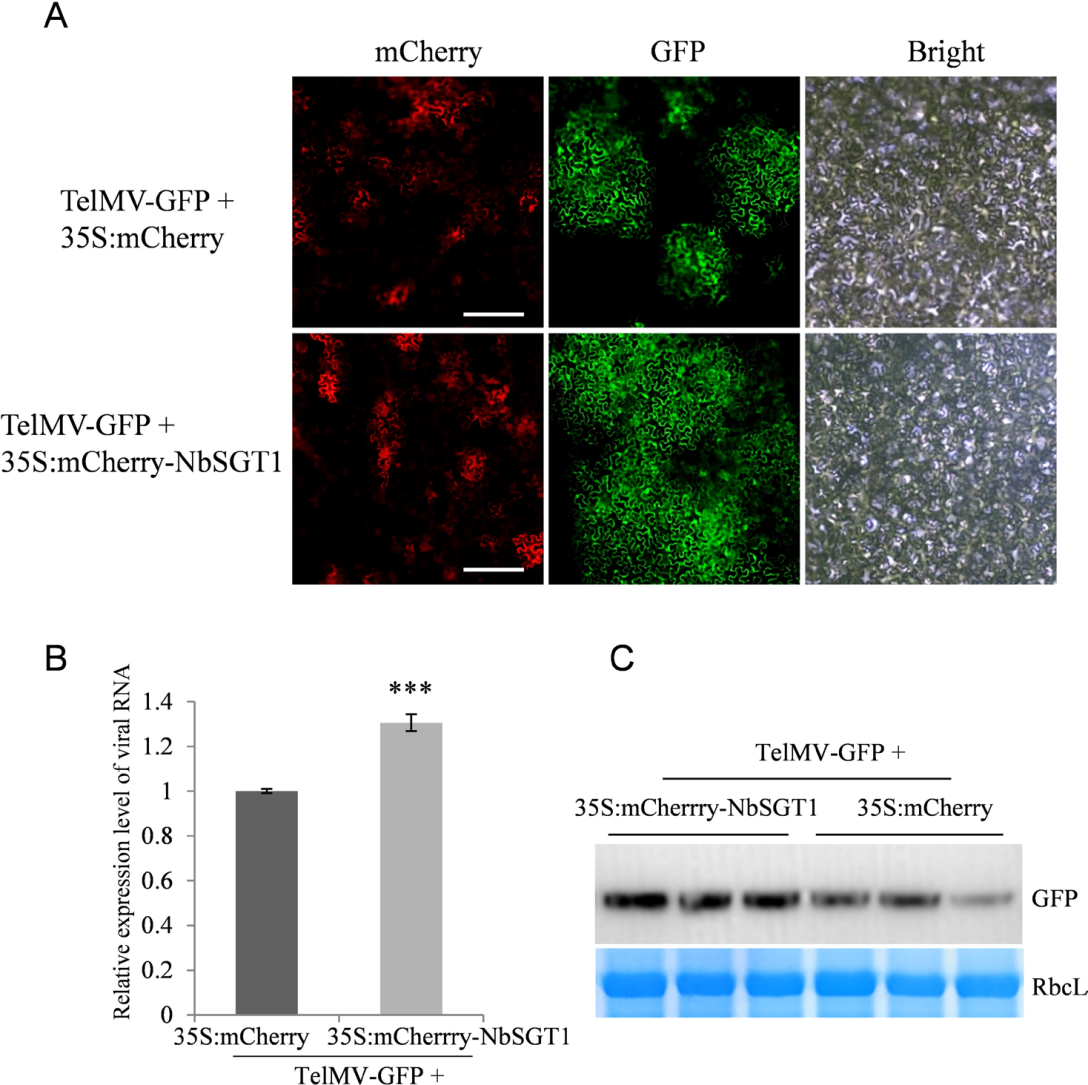
Transient overexpression of NbSGT1 promotes telosma mosaic virus (TelMV) infection in plants. (A) Fluorescent microscope images of the agroinfiltrated leaf inoculated by TelMV‐GFP, along with mCherry or mCherry‐NbSGT1 expressing constructs at 72 h post‐inoculation (hpi). Bars, 200 μm. (B) Quantification of viral RNA accumulation level by reverse transcription‐quantitative PCR analysis at 72 hpi. (C) Immunoblotting analysis of GFP accumulated in the local leaves of TelMV‐GFP‐infected plants at 72 hpi. The Coomassie Brilliant Blue‐stained RbcL serves as a loading control. Error bars denote standard error and Student's *t* test was applied to determine statistical significance (***p < 0.001). All experiments were repeated with similar results.

We further investigated the effects of transient overexpression of *NbSGT1* in TelMV infection in *N. benthamiana* plants. To this end, we constructed an mCherry‐tagged NbSGT1‐expression plasmid under the control of 35S promoter (termed 35S:mCherry‐NbSGT1). The mCherry‐expression vector (35S:mCherry) served as the control. Next, we coinfiltrated the infectious clone TelMV‐GFP with either 35S:mCherry‐NbSGT1 or 35S:mCherry and monitored the viral infection using a fluorescent microscope. At 72 hpi, leaves expressing 35S:mCherry‐NbSGT1 displayed a much stronger green fluorescence intensity compared to the control samples (Figure 4A), suggesting transient overexpression of *NbSGT1* promoted TelMV‐GFP infection. To quantify the virus accumulation level, we conducted RT‐qPCR analysis at 72 hpi. The results indicated a significant increase in viral RNA in the leaves overexpressing *NbSGT1* (Figure 4B). Consistently, western blot analysis showed more intense bands corresponding to the GFP in leaf samples overexpressing *NbSGT1* compared to the control samples (Figure 4C). Taken together, these data revealed that transient overexpression of *NbSGT1* promotes TelMV‐GFP infection in plants.

### 2.7 K217, I227 and E332 of HC‐Pro Are Probably the Residues for Its Interaction With NbSGT1

**FIGURE 5:**
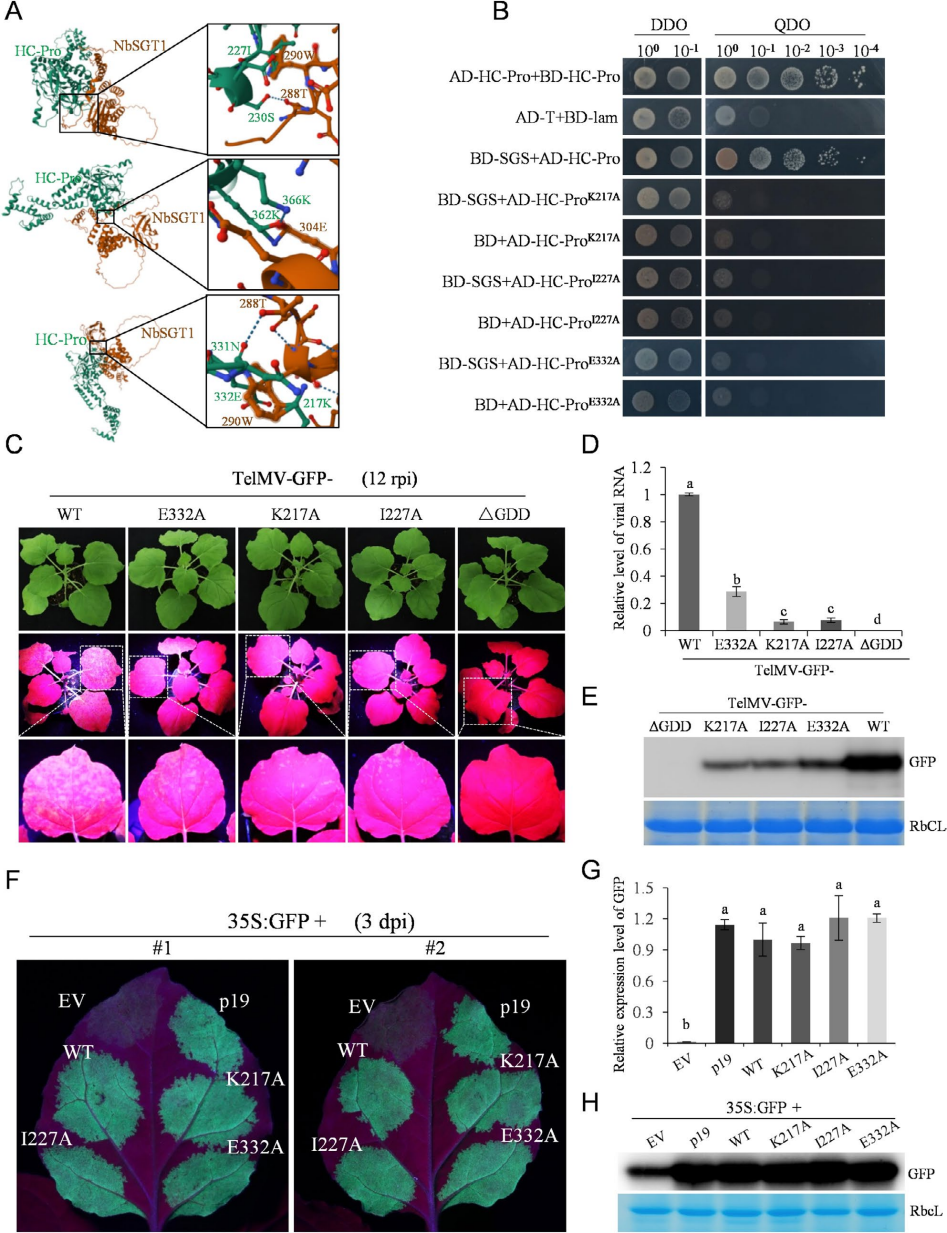
Identification of key residues in HC‐Pro for its interactions with NbSGT1, along with the effects of mutations in these residues on viral systemic infection. (A) Top three predictions of the NbSGT1–HC‐Pro interaction sites by the DMFold server. (B) Yeast two‐hybrid analysis of the interaction between the SGS domain of NbSGT1 and the three HC‐Pro point mutants. (C) Representative images showing the systemic infection of wild‐type (WT) or mutant telosma mosaic virus (TelMV) in *Nicotiana benthamiana* plants at 12 days post‐rub‐inoculation (rpi). (D) Reverse transcription‐quantitative PCR (RT‐qPCR) analysis of the mRNA of viral CP level from systemic leaf of *N. benthamiana* plants inoculated by WT and mutant TelMV. (E) Immunoblotting analysis of GFP from systemic leaf of *N. benthamiana* plants inoculated by WT and mutant TelMV. The Coomassie Brilliant Blue (CBB)‐stained RbcL serves as a loading control. Bars with different lowercase letters are significantly different (one‐way ANOVA). All experiments were repeated with similar results. (F) GFP‐silencing assay in *N. benthamiana* leaves. GFP‐expression construct was co‐expressed with vectors expressing WT HC‐Pro or mutated HC‐Pro and monitored under UV light at 3 days post‐inoculation (dpi). EV, empty vector; p19, RNA silencing suppressor P19 from tomato bushy stunt virus (TBSV). (G) Quantification of GFP mRNA accumulation level by RT‐qPCR analysis at 3 dpi. Bars with different lowercase letters are significantly different (one‐way ANOVA). All experiments were repeated with similar results. (H) Western blot analysis of GFP expression and HC‐Pro expression using anti‐GFP and anti‐Myc. The CBB‐stained RbcL serves as a loading control.

As NbSGT1 interacts with HC‐Pro and promotes TelMV infection, we aimed to examine whether the NbSGT1–HC‐Pro interaction is essential for such enhanced viral infection. First, we needed to determine the key amino acid residues in HC‐Pro that interact with NbSGT1. To this end, we employed DMFold, a deep learning‐based approach to protein complex structure and function prediction built on multiple sequence alignments. The DMFold server identified three potential interaction sites within HC‐Pro: K217, I227 and E332 (Figure 5A). Y2H analysis showed that substituting these residues with alanine abolished interactions with the SGS domain of NbSGT1 (Figure 5B), suggesting K217, I227 and E332 might be the critical residues in HC‐Pro for its interaction with NbSGT1.

### 2.8 The Mutant Virus Carrying K217A, I227A or E332A Reduces Viral Infection, Without Affecting the RSS Activity of HC‐Pro

To determine if abolishing NbSGT1‐HC‐Pro interaction has an impact on viral infection, we performed infectivity assays using mutant viruses carrying the K217A, I227A or E332A mutations in *N. benthamiana* plants. Plants inoculated with TelMV‐GFP carrying the K217A, I227A or E332A mutations exhibited largely reduced viral infections. This was illustrated by the largely reduced green fluorescence observed in the upper non‐inoculated leaves under UV light at three different time points: 6, 9 and 12 rpi (Figure 5C; Figure S5). Additionally, RT‐qPCR and western blot analyses were conducted to detect the viral RNA and protein in the systemic leaves. Compared to plants infected with WT virus, those inoculated with TelMV‐K217A, I227A or E332A exhibited significantly lower viral RNA levels at 12 rpi (Figure 5D). Western blot analysis also showed that less GFP accumulated in the systemic leaves of plants inoculated with the mutant viruses at 12 rpi (Figure 5E).

We next investigated whether the three HC‐Pro mutants might compromise its intrinsic activity as a silencing suppressor. To this end, we constructed a Myc‐tagged plant expression binary vector for the wild‐type HC‐Pro and the three HC‐Pro mutants (K217A, I227A or E332A). These were then subjected to a GFP‐silencing assay to analyse RSS activity. At 3 dpi, leaf patches co‐infiltrated with K217A, I227A or E332A exhibited green fluorescence comparable to that observed with wild‐type HC‐Pro (Figure 5F). This suggests that these three HC‐Pro mutants did not compromise its intrinsic activity as a silencing suppressor. RT‐qPCR indicated no significant difference in GFP mRNA levels between the WT and mutant HC‐Pro samples (Figure 5G). Additionally, western blot analysis of GFP demonstrated similar GFP levels between the WT and mutant HC‐Pro samples (Figure 5H). Finally, we did not observe any noticeable differences in green fluorescence between the WT and mutant HC‐Pro samples, even at 5 or 7 dpi (Figure S6). Collectively, these data demonstrate that the mutant viruses carrying K217A, I227A or E332A showed reduced infectivity without affecting the RSS activity of HC‐Pro.

### 2.9 NbSGT1 Enhances the RSS Activity of HC‐Pro

**FIGURE 6:**
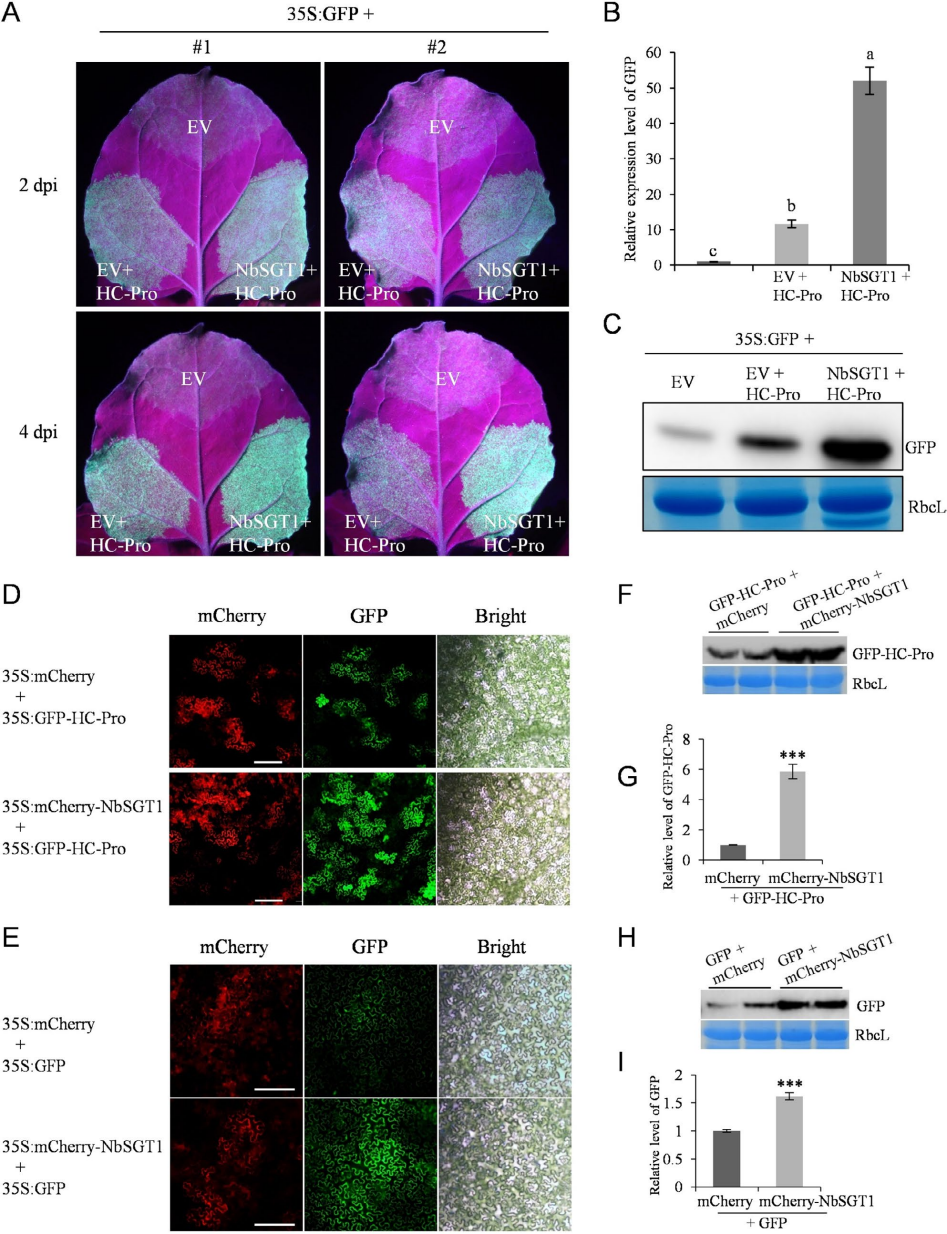
NbSGT1 promotes the RNA silencing suppression (RSS) activity of HC‐Pro, and enhances foreign gene expressions at both the protein and mRNA levels. (A) GFP‐expression construct (35S:GFP) was co‐expressed with vectors expressing empty vector (EV), EV/HC‐Pro or NbSGT1/HC‐Pro and monitored under UV light at 2 and 4 days post‐infiltration (dpi). EV, empty vector. (B) Reverse transcription‐quantitative PCR (RT‐qPCR) analysis of the level of GFP mRNA from co‐infiltrated leaf patches shown in A at 4 dpi. Different lowercase letters above bars indicate significant differences (one‐way ANOVA). (C) Western blot analysis of GFP from co‐infiltrated leaf patches at 4 dpi. (D) Co‐expression of 35S:GFP‐HC‐Pro with 35S:mCherry‐NbSGT1 or 35S:mCherry at *Nicotiana benthamiana* leaves. Leaf epidermal cells were observed under a fluorescence microscope at 84 h post‐infiltration (hpi). Bars, 200 μm. (E) Co‐expression of 35S:GFP with 35S:MCherry‐NbSGT1 or 35S:mCherry at *N. benthamiana* leaves. Leaf epidermal cells were observed under a fluorescent microscope at 84 hpi. Bars, 200 μm. (F) Immunoblotting analysis of GFP‐HC‐Pro abundance from co‐infiltrated leaf patches shown in (D). (G) Quantitative PCR analysis of the level of GFP‐HC‐Pro mRNA from co‐infiltrated leaf patches shown in (D). (H) Immunoblotting analysis of GFP abundance from co‐infiltrated leaf patches shown in E. (I) RT‐qPCR analysis of the level of GFP mRNA from co‐infiltrated leaf patches shown in (E). Error bars in (G) and (I) denote standard error and Student's *t* test was applied to determine statistical significance (***p < 0.001). All experiments were repeated with similar results.

Given that the potyviral HC‐Pro has been well recognised as an RSS in plants, the potential effect of NbSGT1 on its RSS activity was investigated using the classic GFP silencing assay in *N. benthamiana* plants. At 2 dpi, leaf patches coinfiltrated with a mixture of *A. tumefaciens* expressing HC‐Pro and empty vector (EV) displayed noticeable green fluorescence due to the RSS activity of HC‐Pro. Notably, leaf patches coexpressing HC‐Pro and NbSGT1 exhibited an increased intensity of green fluorescence (Figure 6A), suggesting that NbSGT1 may enhance the RSS activity of HC‐Pro. Additionally, such enhancement was observed to persist at 4 dpi (Figure 6A). RT‐qPCR and western blot were conducted to measure the mRNA and protein levels of GFP in the infiltrated patches at 4 dpi. The results demonstrated that mRNA and protein levels of GFP were higher in patches coinfiltrated with 35S:NbSGT1/HC‐Pro compared to those with EV/HC‐Pro (Figure 6B,C). Together, these findings suggest that NbSGT1 promotes the RSS activity of HC‐Pro in *N. benthamiana* plants.

### 2.10 NbSGT1 Promotes Foreign Gene Expression at Both the mRNA and Protein Levels in Plants

Given that NbSGT1 interacts with HC‐Pro, promotes its RSS activity and enhances viral infection, we asked if NbSGT1 promotes HC‐Pro expression in plants. To this end, we fused NbSGT1 and HC‐Pro with mCherry and GFP tags, respectively, and the resulting constructs 35S:mCherry‐NbSGT and 35S:GFP‐HC‐Pro were transiently co‐expressed in the leaves of *N. benthamiana* plants. Interestingly, under a fluorescent microscope, we observed much stronger green fluorescence in leaves co‐expressing GFP‐HC‐Pro with mCherry‐NbSGT1 than with the control mCherry (Figure 6D), suggesting NbSGT1 promotes GFP‐HC‐Pro expression in plant cells. Unexpectedly, in our control experiment, where we co‐expressed GFP with mCherry‐NbSGT1, we also observed stronger green fluorescence (Figure 6E). This indicates that NbSGT1 may also promote GFP expression in plant cells. Western blot analyses confirmed that the protein levels of GFP and GFP‐HC‐Pro were indeed higher in the presence of NbSGT1 (Figure 6F,H).

Next, we aimed to test whether the mRNA levels of GFP and GFP‐HC‐Pro were upregulated upon the expression of NbSGT1. To this end, total RNA was extracted and subjected to RT‐qPCR analysis. The results indicated a significant upregulation of mRNA levels of both GFP and GFP‐HC‐Pro when co‐expressed with NbSGT1 (Figure 6G,I). Overall, these results demonstrate that NbSGT1 promotes heterologous gene expression at both the protein and mRNA levels.

SGT1 is well‐recognised as a molecular cochaperone, and the SGT1–RAR1–HSP90 molecular chaperone complex plays a significant role in diverse biological signalling processes, including development and disease resistance (Seo et al. 2008). We investigated whether NbRAR1 and NbHSP90 could promote the gene expression of GFP by co‐expression experiments, as described earlier. The results from western blot and RT‐qPCR analyses revealed that NbRAR1 did not promote GFP gene expression at either the protein or mRNA level (Figure S7A,D). In contrast, NbHSP90 indeed increased the protein level of GFP (Figure S7B). However, it did not promote the mRNA level of GFP in plant cells (Figure S7E). These results suggest the molecular cochaperone NbSGT1 may have a novel role in regulating gene expression at the mRNA level.

### 2.11 NbSGT1 May Act as an Endogenous Suppressor of RNA Silencing

**FIGURE 7:**
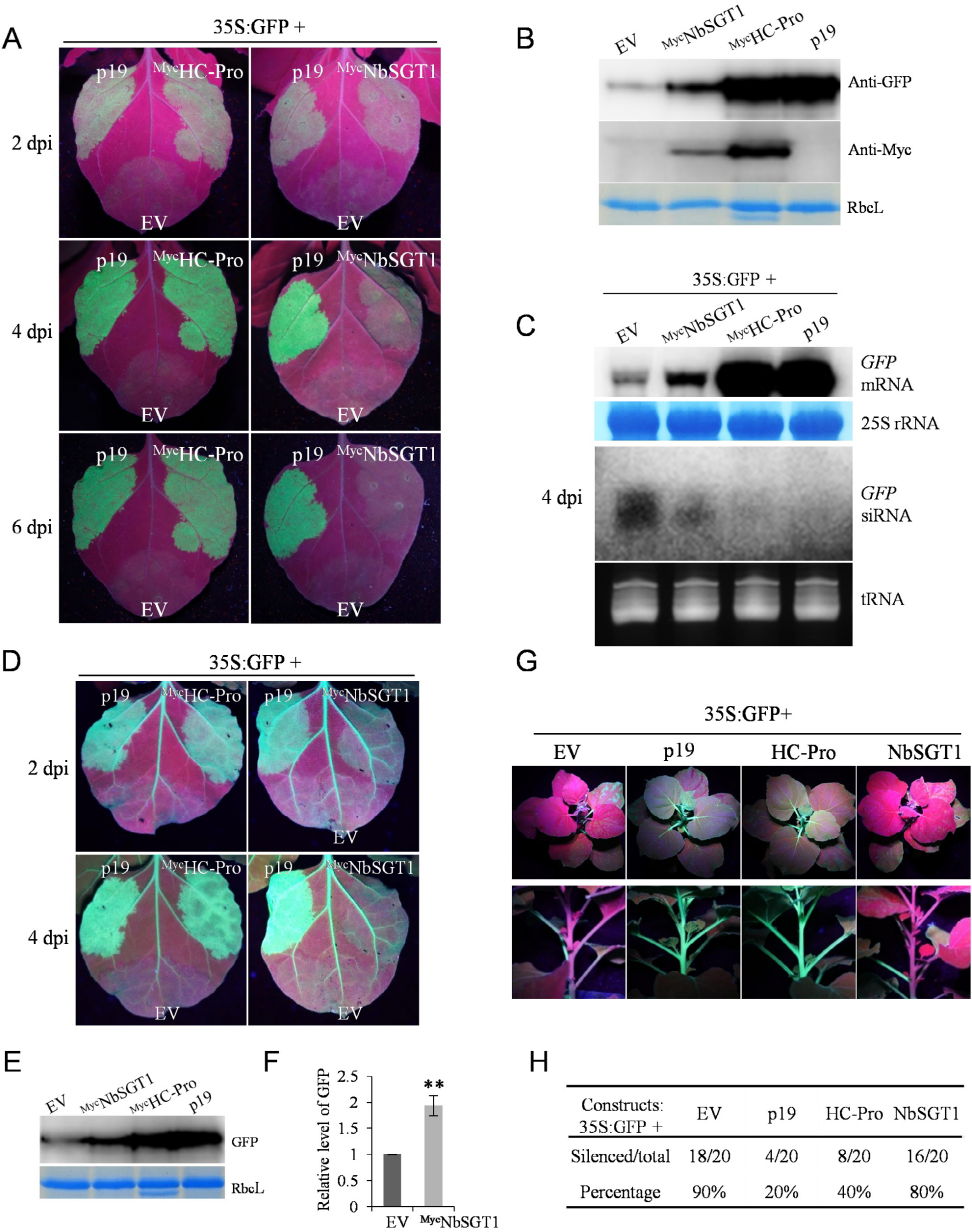
NbSGT1 acts as an endogenous suppressor of RNA silencing, inhibiting local but not systemic RNA silencing in plants. (A) Leaves of wild‐type (WT) *Nicotiana benthamiana* plants agro‐infiltrated with *Agrobacterium tumefaciens* carrying 35S:GFP together with agrobacteria carrying 35S:EV, 35S:p19, 35S:^Myc^HC‐Pro or 35S:^Myc^NbSGT1. Leaf pictures were taken under UV light at 2, 4 and 6 days post‐infiltration (dpi). (B) Immunoblotting analysis of GFP, ^Myc^HC‐Pro and ^Myc^NbSGT1 abundance from co‐infiltrated leaf patches shown in (A) at 4 dpi. (C) Northern blot analysis for the detection of GFP mRNA and GFP transcript‐derived small interfering RNAs (siRNAs) in agroinfiltrated leaves shown in (A) at 4 dpi. Ethidium bromide staining of 25S ribosomal RNA (rRNA) and transfer RNA (tRNA) were used as the loading controls for northern blot assays of *GFP* mRNA transcripts and *GFP*‐derived siRNAs, respectively. (D) Leaves of *N. benthamiana* 16c plants agro‐infiltrated *A. tumefaciens* carrying 35S:GFP together with agrobacteria carrying 35S:EV, 35S:p19, 35S:^Myc^HC‐Pro or 35S:^Myc^NbSGT1. Leaf pictures were taken under UV light at 2 and 4 dpi. (E) Immunoblotting analysis of GFP, ^Myc^HC‐Pro, and ^Myc^NbSGT1 abundance from co‐infiltrated leaf patches shown in (D) at 4 dpi. (F) Reverse transcription‐quantitative PCR analysis of the level of GFP mRNA from co‐infiltrated leaf patches shown in (E) at 4 dpi. (G) Representative *N. benthamiana* 16c plants infiltrated with *A. tumefaciens* carrying 35S:GFP together with *A. tumefaciens* carrying 35S:EV, 35S:p19, 35S:^Myc^HC‐Pro or 35S:^Myc^NbSGT1. Pictures of the upper part of leaves and stems of *N. benthamiana* 16c plants were taken under UV light at 26 dpi. (H) Efficiency of systemic silencing of *GFP* in *N. benthamiana* 16c plants at 26 dpi. Error bars denote standard error and Student's *t* test was applied to determine statistical significance (**p < 0.05). All experiments were repeated with similar results.

Next, we tested whether NbSGT1 plays a role in PTGS using the broadly used *Agrobacterium*‐mediated transient expression system (Johansen and Carrington 2001). The leaves of WT *N. benthamiana* plants were co‐infiltrated with *A. tumefaciens* cultures harbouring 35S:GFP, along with *A. tumefaciens* carrying Myc‐tagged NbSGT1 (35S:^Myc^NbSGT1). GFP fluorescence intensity was monitored over a period of 6 days. As expected, the positive controls (35S:p19 or 35S:HC‐Pro) demonstrated high levels of GFP signal at all three time points tested (2, 4 and 6 dpi). Notably, at 2 dpi, patches infiltrated with 35S:^Myc^NbSGT1 displayed noticeable GFP fluorescence, followed by a gradual increase at 4 dpi and a nearly complete loss of GFP signal at 6 dpi (Figure 7A). In contrast, patches infiltrated with the negative control (empty vector, 35S:EV) displayed a very weak GFP signal at 4 dpi and the green fluorescence nearly disappeared at 6 dpi (Figure 7A). These results suggest that NbSGT1 suppressed PTGS in plants.

Additionally, western blot and northern blot were performed to assess the protein and mRNA levels of GFP in the infiltrated patches at 4 dpi. Western blot analysis showed that GFP protein was higher in the patches co‐infiltrated with 35S:^Myc^NbSGT1 compared to those with the EV control (Figure 7B). Also, the successful expression of Myc‐NbSGT1 was confirmed by western blot using anti‐Myc antibody (Figure 7B; Figure S8). Similarly, northern blot results revealed a higher accumulation of GFP mRNA in the patches treated with 35S:^Myc^NbSGT1 compared to the EV control (Figure 7C).

We examined the accumulation of GFP‐specific small interfering RNAs (siRNAs), which serve as an indicator of RNA silencing, in the infiltrated leaves by northern blot analysis. The results showed that the accumulation of GFP siRNA was lower in the patches infiltrated with 35S:^Myc^NbSGT1 and 35S:GFP compared to the EV control (Figure 7C). Overall, these results revealed that NbSGT1 suppresses RNA silencing in plants.

### 2.12 NbSGT1 Inhibits Local, but Not Systemic, RNA Silencing in Plants

To further confirm that NbSGT1 suppresses local RNA silencing in plants, we conducted transient expression assays in *N. benthamiana* 16c plants. GFP expression was monitored in the infiltrated leaves. In the patches co‐agroinfiltrated with 35S:GFP plus 35S:^Myc^NbSGT1 showed clear green fluorescence compared to the EV samples at both 2 and 4 dpi (Figure 7D). Western blot and RT‐qPCR analyses indicated that both the protein and mRNA levels of GFP in the patches infiltrated with 35S:NbSGT1 were higher than those of EV (Figure 7E,F). Together, these results demonstrated that NbSGT1 suppresses local RNA silencing in *N. benthamiana* plants.

Next, we investigated whether NbSGT1 inhibits systemic RNA silencing in plants. GFP expression was monitored in the upper non‐infiltrated leaves of *N. benthamiana* 16c plants infiltrated with 35S:GFP plus 35S:NbSGT1, 35S:EV, 35S:p19 or 35S:HC‐Pro over a period of 26 days. At 26 dpi, almost all plants (18/20) infiltrated with 35S:EV and 35S:GFP exhibited red fluorescence under UV light in the major and minor veins of upper noninfiltrated leaves (Figure 7G,H), suggesting infiltration of 35S:GFP in the local leaf triggered systemic silencing of *GFP* in 16c plants. As p19 and HC‐Pro are known to suppress systemic RNA silencing in plants, these two viral proteins were used as the positive controls. As shown in Figure 7G, both the upper emerging leaf tissue and stems of many plants infiltrated with these positive controls remained green under UV light, indicating p19 and HC‐Pro were effective in suppressing systemic silencing of *GFP*. In contrast, in most plants (16/20) infiltrated with 35S:GFP and 35S:NbSGT1, both the upper emerging leaf and the stem turned red at 26 dpi (Figure 7G,H), suggesting NbSGT1 was unable to suppress systemic GFP silencing in plants.

Together, these results indicate that NbSGT1 suppresses local, but not systemic, RNA silencing in plants.

### 2.13 NbSGT1 Downregulates the Expression of Key Components of the RNA Silencing Pathway

**FIGURE 8:**
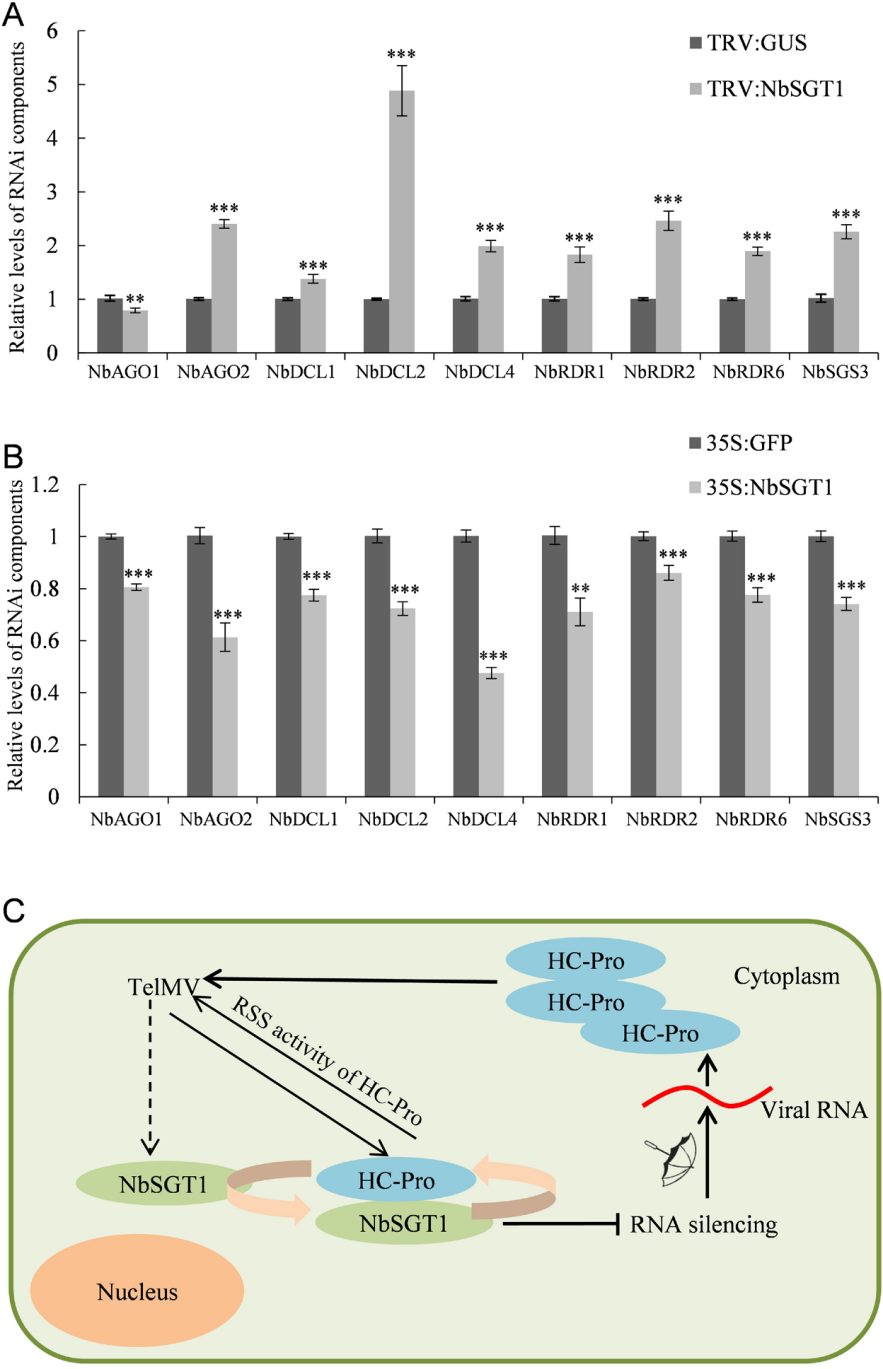
NbSGT1 suppresses the expression of key components of the RNA silencing pathway in plants and the proposed model for NbSGT1–telosma mosaic virus (TelMV) interaction. (A) Relative expression level of key components of the RNA silencing pathway in the *NbSGT1*‐silenced *Nicotiana benthamiana* plants (TRV:NbSGT1) at 12 days post‐infiltration (dpi). TRV:GUS plants serve as the control. (B) Relative expression level of key components of the RNA silencing pathway in *N. benthamiana* leaves infiltrated with 35S:NbSGT1 or 35S:GFP at 48 h post‐infiltration (hpi). Error bars denote standard error and Student's *t* test was applied to determine statistical significance (**p < 0.05; ***p < 0.001). (C) Proposed model for the role of NbSGT1 in promoting potyviral infection. First, NbSGT1 interacts with HC‐Pro, enhancing its RNA silencing suppression (RSS) activity, thereby promoting TelMV infection. Additionally, NbSGT1 may function as an endogenous suppressor of RNA silencing (ESR), negatively regulating RNA silencing and facilitating potyviral infection. The dashed line arrowhead indicates the potential inducible gene expression of *NbSGT1* following viral infection.

To investigate whether NbSGT1 regulates the gene expression of key components of the RNA silencing pathway, such as *AGOs*, *DCLs*, *RDRs* and *SGS3*, the *NbSGT1*‐silenced plants (TRV‐NbSGT1) were examined for the mRNA levels of these RNAi components. RT‐qPCR analyses revealed that *NbAGO2*, *NbDCL1*, *NbDCL2*, *NbDCL4*, *NbRDR1*, *NbRDR2*, *NbRDR6* and *NbSGS3* were all significantly up‐regulated in the *NbSGT1*‐silenced plants (Figure 8A). Among these, *NbDCL2* showed an increase of 4.9‐fold, *NbAGO2*, *NbRDR2* and *NbSGS3* also increased by 2.4‐fold, 2.5‐fold and 2.3‐fold, respectively. These data suggest that silencing of *NbSGT1* promotes the expression of *DCLs*, *RDRs* and *NbSGS3* in *N. benthamiana* plants.

Next, we transiently overexpressed *NbSGT1* in *N. benthamiana* plants and conducted RT‐qPCR to measure the transcription levels of these key RNAi components. At 48 dpi, we observed a significant decrease in the mRNA levels of all tested RNAi components, suggesting transient expression of *NbSGT1* suppressed the expression of *AGOs*, *DCLs*, *RDRs* and *SGS3* in *N. benthamiana* plants (Figure 8B). Altogether, these results indicate that NbSGT1 negatively regulates the expression of key components of the RNA silencing pathway.

## 3 Discussion

SGT1 is well‐known as a molecular co‐chaperone and plays various roles in plant development and immunity (Meldau et al. 2011; Zhang et al. 2024). Recent studies also revealed that SGT1 is involved in plant virus infection, mainly as a co‐chaperone or an actor in disease resistance pathways. Herein, we report the evidence that the molecular co‐chaperone SGT1 has a new role acting as a host ESR and is recruited by a potyvirus through HC‐Pro interaction for robust viral infection. We propose a model for the role of NbSGT1 in promoting potyviral infection, highlighting (1) the pivotal role of the HC‐Pro‐NbSGT1 interaction in potyviral infection and (2) the endogenous suppression of RNA silencing (Figure 8C).

### 3.1 NbSGT1 Interacts With HC‐Pro

TelMV is an emerging plant virus that infects passion fruit, but research on TelMV–host interactions is still in its infancy. In this study, we used the multifunctional viral protein HC‐Pro as the bait protein and identified the potential interacting host proteins, including the molecular co‐chaperone NbSGT1 (Table 1; Figure 1). To our knowledge, this is the first report on host protein candidates involved in TelMV infection, providing valuable information for TelMV–host interactions. Furthermore, the NbSGT1‐HC‐Pro interactions were confirmed by Y2H, BiFC and co‐immunoprecipitation analyses (Figure 2). Using Y2H and BiFC, the domain of NbSGT1 responsible for its interaction with HC‐Pro was mapped to the SGS domain (Figure 2). The SGS domain has been reported to mediate interactions between SGT1 and other previously identified SGT1‐interacting partners, including HSC70, Cyr1/Cdc35, S100A6, Prf, Bs2, MLA1 and MLA6 (Dubacq et al. 2002; Nowotny et al. 2003; Noël et al. 2007; Wang et al. 2022; Chapman et al. 2021). Future experiments should be devoted to determining the responsible domain in HC‐Pro for its interaction with NbSGT1.

Given that TelMV HC‐Pro is an RSS (Wang et al. 2024) and interacts with NbSGT1 (Figure 2), we examined if NbSGT1 affects the RSS activity of HC‐Pro. Co‐expression of HC‐Pro and NbSGT1 in plants significantly improved the RSS activity of HC‐Pro in a GFP‐silencing assay (Figure 6). This improvement could be explained, at least partially, by the increased protein levels of HC‐Pro in the presence of NbSGT1 (Figure 6F). To date, dozens of host proteins involved in various cellular pathways have been reported to interact with HC‐Pro (Hýsková et al. 2024). However, few of them regulate the RSS activity of HC‐Pro. In contrast to our study, ZmVDE and ZmTGL have been reported to attenuate the RSS activities of HC‐Pros (Chen et al. 2017; Xu et al. 2022). Furthermore, using three HC‐Pro mutants with compromised capacity for interacting with the SGS domain of NbSGT1, we showed that this interaction is essential for viral infection (Figure 5). We are also interested in knowing if the NbSGT1–HC‐Pro interaction is pivotal for the infection by other potyviruses. The specific mode of HC‐Pro–NbSGT1 interaction and the detailed molecular mechanism by which they affect viral infection needs to be further investigated.

### 3.2 NbSGT1 Is a Negative Regulator of RNA Silencing

SGT1 is well recognised as a molecular cochaperone involved in development and immunity. Herein, we provide several pieces of evidence demonstrating that NbSGT1 acts as an endogenous suppressor of RNA silencing in plants. Firstly, in addition to its classic role as a molecular cochaperone that enhances protein levels, NbSGT1 also increased the transcription level of foreign genes (Figure 6). Secondly, NbSGT1 suppressed RNA silencing in both WT and 16c *N. benthamiana* plants in the *Agrobacterium* infiltration assay (Figure 7). Thirdly, NbSGT1 inhibited local but not systemic RNA silencing in *N. benthamiana* plants (Figure 7). Lastly, NbSGT1 significantly downregulated the expression of key components of the RNA silencing pathway, including *AGOs*, *DCLs*, *RDRs* and *SGS3* (Figure 8A,B). Nevertheless, the exact mechanism by which NbSGT1 suppresses RNA silencing remains unclear and requires further investigation. Is it integrated into the classical RNA silencing suppression pathway, or does it operate through a novel mechanism distinct from those currently known?

Recent advances have revealed intriguing noncanonical roles of classic molecular chaperones, functioning independently of their chaperone activity. For example, Hsc70‐4 can specifically bind to dsRNA and mediates its internalisation in *Drosophila*, without relying on its chaperone function (Fletcher et al. 2025). Our data also suggest that the plant molecular chaperone HSP70, but not HSP90, can promote foreign gene expression at the mRNA level in plants (Figure S7E,F). Along with our findings regarding the novel role of NbSGT1 as an ESR, it is becoming increasingly clear that some molecular chaperones exhibit additional functions beyond their traditional chaperone activities.

Our findings provide evidence that the molecular co‐chaperone SGT1 has a new function acting as a host ESR, and is recruited by a potyvirus for infection. This work provides a new perspective on how SGT1 regulates protein homeostasis beyond its traditional role as a molecular co‐chaperone. It also lays the groundwork for several future studies, including (1) the structural and functional properties of SGT1 in RNA silencing suppression; (2) the molecular mechanisms underpinning the recruitment of SGT1 by plant viruses for efficient infection; (3) whether HC‐Pro requires SGT1 to fulfil its RSS function; and lastly, and (4) the mechanism by which SGT1 suppresses RNA silencing.

## 4 Experimental Procedures

### 4.1 Plant and Growth Conditions

*Nicothiana benthamiana* plants were grown in a growth chamber. The growth conditions were set at 65% relative humidity at 24°C for 16 h of light and 22°C for 8 h of darkness.

### 4.2 Virus and Plant Inoculation

The infectious clone of pTelMV‐GFP was from our lab (Gou et al. 2023). For assessing the infectivity of TelMV infectious clones and evaluating the impact of *NbSGT1* overexpression on viral infection, *N. benthamiana* was agroinfiltrated with TelMV‐GFP. To investigate the effects of TRV‐mediated *NbSGT1* silencing on viral infection, we employed rub‐inoculation.

### 4.3 Plasmid Construction

The viral infectious clones pTelMV 2 × StrepHCPro‐GFP were constructed using pTelMV‐GFP as the template. Two fragments were amplified with primer pairs P1‐F/strepHCPro‐1‐R and strepHCPro‐2‐F/N1‐R (Table S1), respectively, followed by overlapping PCR using primers P1‐F and N1‐R to generate the HCPro fragment with an added Twin‐Strep tag. The fragment was double digested with PstI and SalI enzymes and subsequently ligated into the predigested backbone vector pTelMV‐GFP. Mutant viruses were generated via overlapping PCR using pTelMV‐GFP as the template.

For Y2H, BiFC and co‐immunoprecipitation assays, the full‐length coding sequences (CDS) of *NbSGT1* and *HC‐Pro* were cloned into the entry vector pDONR221 via Gateway cloning technology and subsequently recombined into the corresponding expression vectors (Qin et al. 2024). For RNAi‐mediated silencing of *NbSGT1*, partial coding region sequences of *NbSGT1* were inserted into the p2300s‐intron vector (Hu et al. 2025). For transient co‐expression experiments, the full‐length CDS of *NbSGT1*, *NbRAR1*, *NbHSP90* and *NbHSP70* were cloned into pCaM‐mCherry to drive protein expression. For the RNA silencing suppression assay, the full‐length CDS of *NbSGT1* was cloned into pCaMterX to generate the plasmid pCaM‐Myc‐NbSGT1. All constructed plasmids were validated by Sanger DNA sequencing.

### 4.4 LC–MS/MS Analyses

*Nicotiana benthamiana* leaves were inoculated with TelMV‐GFP (OD_600_ = 1.0) expressing the 2 × Strep‐tagged HC‐Pro protein. At 15 dpi, protein was extracted from the upper systemic leaves showing viral infection. The extraction was performed following the previously described method (Hu et al. 2022). Subsequently, the HC‐Pro‐interacting proteins were identified by LC–MS/MS (Beijing Biotech Pack Scientific Co. Ltd.). The raw mass spectrometry data were analysed using MaxQuant (v. 1.6.2.10) and searched against the UniProt protein database (https://www.uniprot.org/) to obtain the protein candidates.

### 4.5 Western Blotting

Total proteins were extracted from *N. benthamiana* leaves using lysis buffer containing 50 mM Tris–HCl (pH 6.8), 50 mM dithiothreitol (DTT), 4% SDS, 10% glycerol, 1% PVP‐40 and 5% PMSF. The mixture was boiled for 10 min and centrifuged at 13,400 *g* for 10 min at 4°C. The supernatant was transferred to a fresh microcentrifuge tube, and 5× SDS loading buffer was added. The protein samples were separated by 10% SDS‐PAGE and subsequently transferred to PVDF membrane. The membrane was blocked with blocking buffer (5% skim milk and 0.1% Tween 20 in phosphate‐buffered saline) at room temperature for 30 min. Subsequently, anti‐GFP or anti‐Myc antibody were used as primary antibodies, and horseradish peroxidase (HRP)‐conjugated anti‐mouse or anti‐rabbit were used as secondary antibody. Protein signals were detected using a chemiluminescence imaging system (JINGYI).

### 4.6 Y2H, BiFC and Co‐Immunoprecipitation

Y2H, BiFC and co‐immunoprecipitation assays were essentially as described (Qin et al. 2024).

### 4.7 RNA Extraction, Northern Blot, siRNA Blot, RT‐PCR and Real‐Time qPCR

Total RNA was extracted from *N. benthamiana* leaves using TRIzol Universal (TIANGEN). For first‐strand cDNA synthesis, total RNA was treated with DNase I to eliminate genomic DNA contamination. First‐strand cDNA was synthesised using the RevertAid first‐strand cDNA synthesis kit (Thermo Fisher Scientific). Real‐time PCR was performed on an Applied Biosystems QuantStudio 5 Real‐Time PCR System (Thermo Fisher Scientific) using the SuperReal PreMix Plus kit (TIANGEN). The *Actin* gene of *N. benthamiana* was used as an internal control for normalisation. The northern and siRNA blots were essentially as described (Hu et al. 2022).

### 4.8 Prediction of Key Amino Acids of Interacting Proteins

The interaction between NbSGT1 and HC‐Pro was predicted using DMFold (https://zhanggroup.org/DMFold/). The top five highest‐scoring models were selected, and their interaction interfaces were analysed via PDBePISA (https://www.ebi.ac.uk/pdbe/pisa/) and Mol* 3D Viewer (https://www.rcsb.org/3d‐view).

### 4.9 VIGS

VIGS was essentially as described (Liu, Schiff, Marathe, and Dinesh‐Kumar 2002; Liu, Schiff, Serino, et al. 2002).

### 4.10 RNA Silencing Suppression Assay and GFP Imaging

RNA silencing suppression assay and GFP imaging were essentially as described (Wang et al. 2024).

## Author Contributions

**Wei Shi:** methodology, investigation, writing – original draft, writing – review and editing, validation, data curation, resources, formal analysis, software, visualization. **Liwen Zhang:** software, writing – original draft, writing – review and editing, formal analysis, validation, resources. **Na Li:** writing – review and editing, validation, writing – original draft, software, formal analysis, resources. **Bei Gou:** writing – original draft, validation, writing – review and editing, software, formal analysis, resources. **Li Qin:** conceptualization, writing – original draft, writing – review and editing, resources. **Wenping Qiu:** conceptualization, resources, formal analysis, validation, writing – review and editing, supervision. **Hongguang Cui:** funding acquisition, writing – original draft, writing – review and editing, conceptualization, project administration, resources, supervision. **Hui Wang:** conceptualization, writing – original draft, writing – review and editing, project administration, resources. **Zhaoji Dai:** conceptualization, funding acquisition, writing – original draft, writing – review and editing, methodology, project administration, supervision, data curation, resources.

## Conflicts of Interest

The authors declare no conflicts of interest.

## Supporting information


**Figure S1:** Amino acid sequence alignment using NbSGT1 sequences from *N. benthamiana* (AAW82048.1), *A. thaliana* (AAL33611.1, AAL33612.1), *G. max* (ACI31549.1, ACI31550.1), *H. vulgare* (AAL33610.1), *O. sativa* (AAF18438.1), *Z. mays* (AKD95366.1), *P. sogdiana* (ATE46012.1), *G. sanguineum* (ABH03408.1), *T. aestivum* (AIX87834.1), *V. pseudoreticulata* (AFM35697.1), *M. hupehensis* (ACR78249.1), *T. intermedium* (ABQ10569.1), *C. moschata* (AZZ86680.1), *S. lycopersicum* (NP_001307150.1), *D. villosum* (AGA16735.1), *F. esculentum* (UNO37372.1), and *Z. jujube* (XP_015878320.1).

**Figure S2:** The phylogenetic tree shows evolutionary relationships among plant SGT1 proteins. Amino acid sequence alignments were performed using the MAFFT algorithm implemented in PhyloSuite, and the tree was constructed using IQ‐TREE with the Maximum Likelihood method. The scale bar represents the number of substitutions per site.

**Figure S3:** Representative images showing NbSGT1‐silenced *N. benthamiana* at 13, 17, and 21 dpi. TRV:PDS and TRV:GUS serve as controls. The right panel shows qRT‐PCR analysis of NbSGT1 silencing efficiency by TRV‐VIGS at the corresponding time points.

**Figure S4:** Confirmation of the presence of TRV by detecting the *CP* gene (upper panel), and the existence of TRV:GUS as well as TRV:NbSGT1 using appropriate primers flanking the inserts (lower panel) in the systemic leaf of *N. benthamiana* in TRV:GUS and TRV:NbSGT1‐inoculated plants, respectively. The *N. benthamiana Actin* gene serves as the internal control.

**Figure S5:** Representative images showing the systemic infection of WT or mutant TelMV in *N. benthamiana* plants at 6 and 9 dpi.

**Figure S6:** RNA silencing suppression analysis of WT and mutated HC‐Pros in *N. benthamiana* plant. GFP‐expression construct was co‐expressed with vectors expressing WT HC‐Pro or mutated HC‐Pros, respectively and monitored under UV light at 3, 5, and 7 dpi, respectively. EV, empty vector; p19, RNA silencing suppressor P19 from tomato bushy stunt virus (TBSV).

**Figure S7:** Effect of NbRAR1, NbHSP90, and NbHSP70 on the expression of GFP at the protein and mRNA levels. (A, B, and C) Immunoblotting analysis of GFP abundance from leaf patches co‐infiltrated by 35S:GFP with 35S:NbRAR1‐mCherry, 35S:NbHSP70‐mCherry, or 35S:NbHSP70‐mCherry. (D, E and F) Quantitative PCR analysis of the level of GFP mRNA from leaf patches co‐infiltrated by 35S:GFP with 35S:NbRAR1‐mCherry, 35S:NbHSP70‐mCherry, or 35S:NbHSP70‐mCherry.

**Figure S8:** Western blot analysis of NbSGT1 expression and HC‐Pro expression using anti‐Myc at different time points (2, 4, and 6 dpi). The Coomassie brilliant blue (CBB)‐stained Rubisco large subunit (RbcL) serves as a loading control.

**Table S1:** Primers used in this study.

## Data Availability

The data that support the findings of this study are available on request from the corresponding author. The data are not publicly available due to privacy or ethical restrictions.
