# Supplementary figures and images for: The Molecular Cochaperone NbSGT1 May Function as an Endogenous Suppressor of RNA Silencing That Is Recruited by a Potyvirus in Infection of Plants
Source: Mol Plant Pathol. 2026 Feb 18;27(2):e70221. doi: 10.1111/mpp.70221 (PMC12916245; doi:10.1111/mpp.70221)

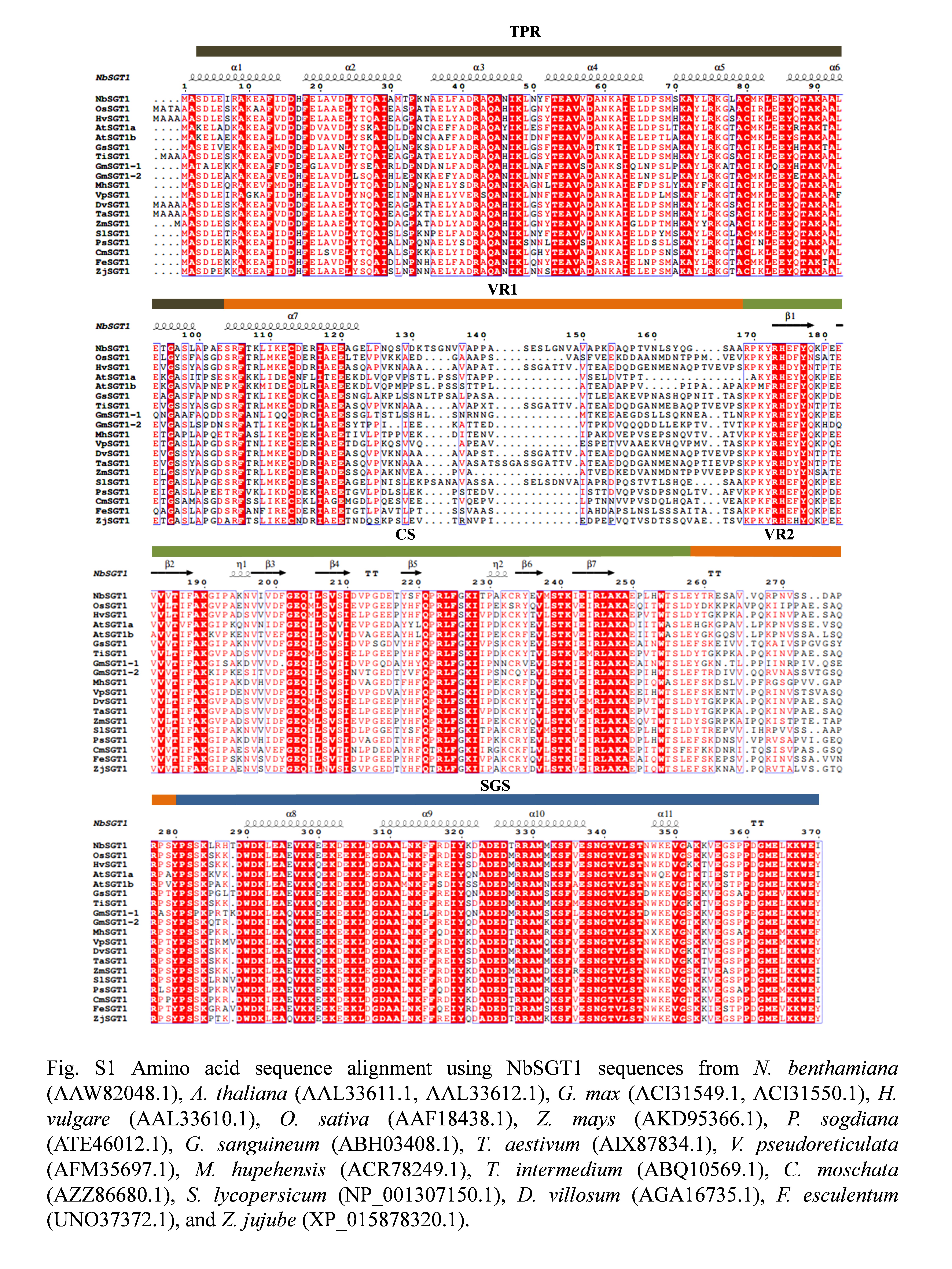

Supplement: Supplementary file 1 — Figure S1: Amino acid sequence alignment using NbSGT1 sequences from N. benthamiana (AAW82048.1), A. thaliana (AAL33611.1, AAL33612.1), G. max (ACI31549.1, ACI31550.1), H. vulgare (AAL33610.1), O. sativa (AAF18438.1), Z. mays (AKD95366.1), P. sogdiana (ATE46012.1), G. sanguineum (ABH03408.1), T. aestivum (AIX87834.1), V. pseudoreticulata (AFM35697.1), M. hupehensis (ACR78249.1), T. intermedium (ABQ10569.1), C. moschata (AZZ86680.1), S. lycopersicum (NP_001307150.1), D. villosum (AGA16735.1), F. esculentum (UNO37372.1), and Z. jujube (XP_015878320.1). [file MPP-27-e70221-s008.jpg]

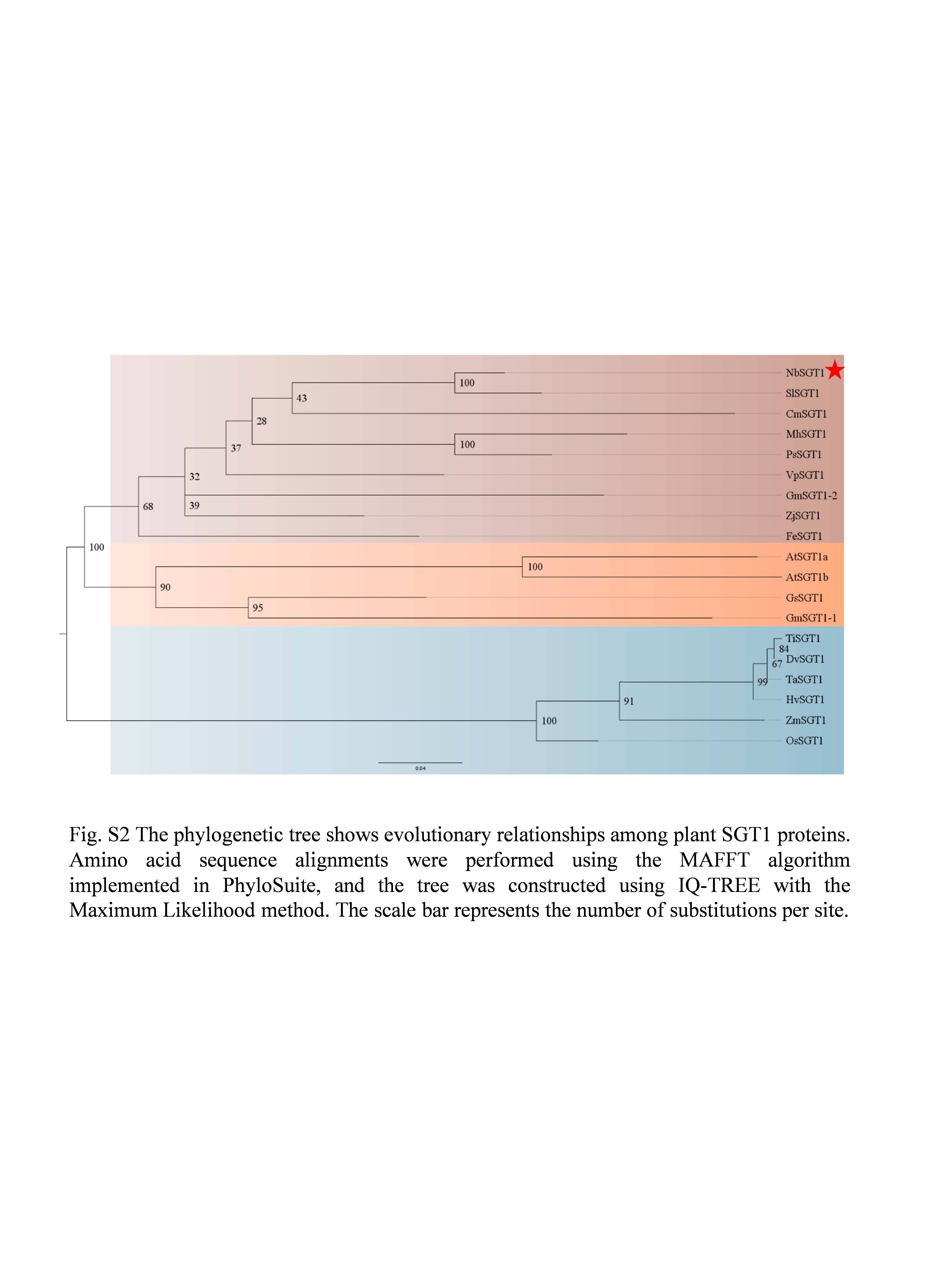

Supplement: Supplementary file 2 — Figure S2: The phylogenetic tree shows evolutionary relationships among plant SGT1 proteins. Amino acid sequence alignments were performed using the MAFFT algorithm implemented in PhyloSuite, and the tree was constructed using IQ‐TREE with the Maximum Likelihood method. The scale bar represents the number of substitutions per site. [file MPP-27-e70221-s005.jpg]

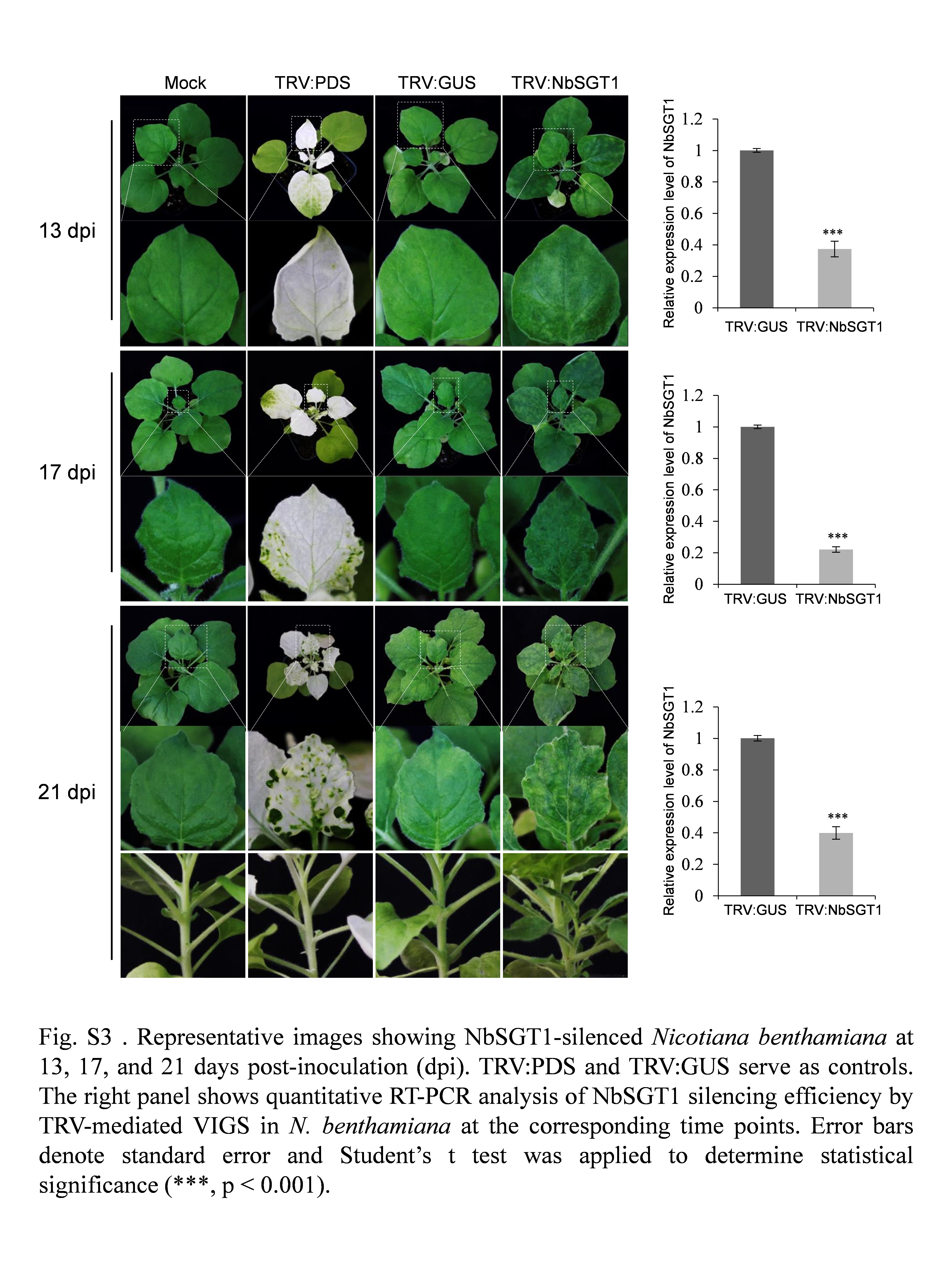

Supplement: Supplementary file 3 — Figure S3: Representative images showing NbSGT1‐silenced N. benthamiana at 13, 17, and 21 dpi. TRV:PDS and TRV:GUS serve as controls. The right panel shows qRT‐PCR analysis of NbSGT1 silencing efficiency by TRV‐VIGS at the corresponding time points. [file MPP-27-e70221-s009.jpg]

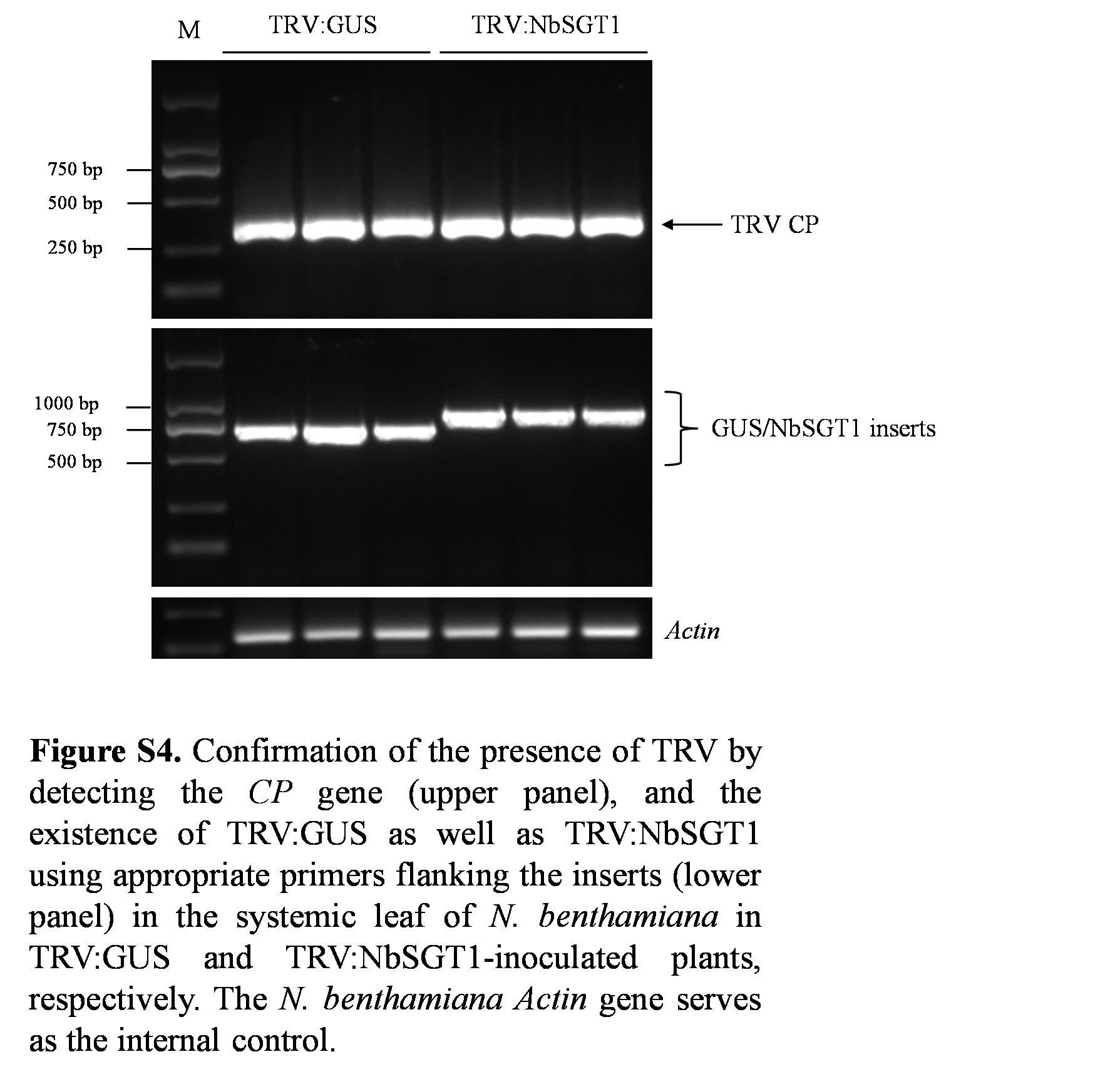

Supplement: Supplementary file 4 — Figure S4: Confirmation of the presence of TRV by detecting the CP gene (upper panel), and the existence of TRV:GUS as well as TRV:NbSGT1 using appropriate primers flanking the inserts (lower panel) in the systemic leaf of N. benthamiana in TRV:GUS and TRV:NbSGT1‐inoculated plants, respectively. The N. benthamiana Actin gene serves as the internal control. [file MPP-27-e70221-s002.jpg]

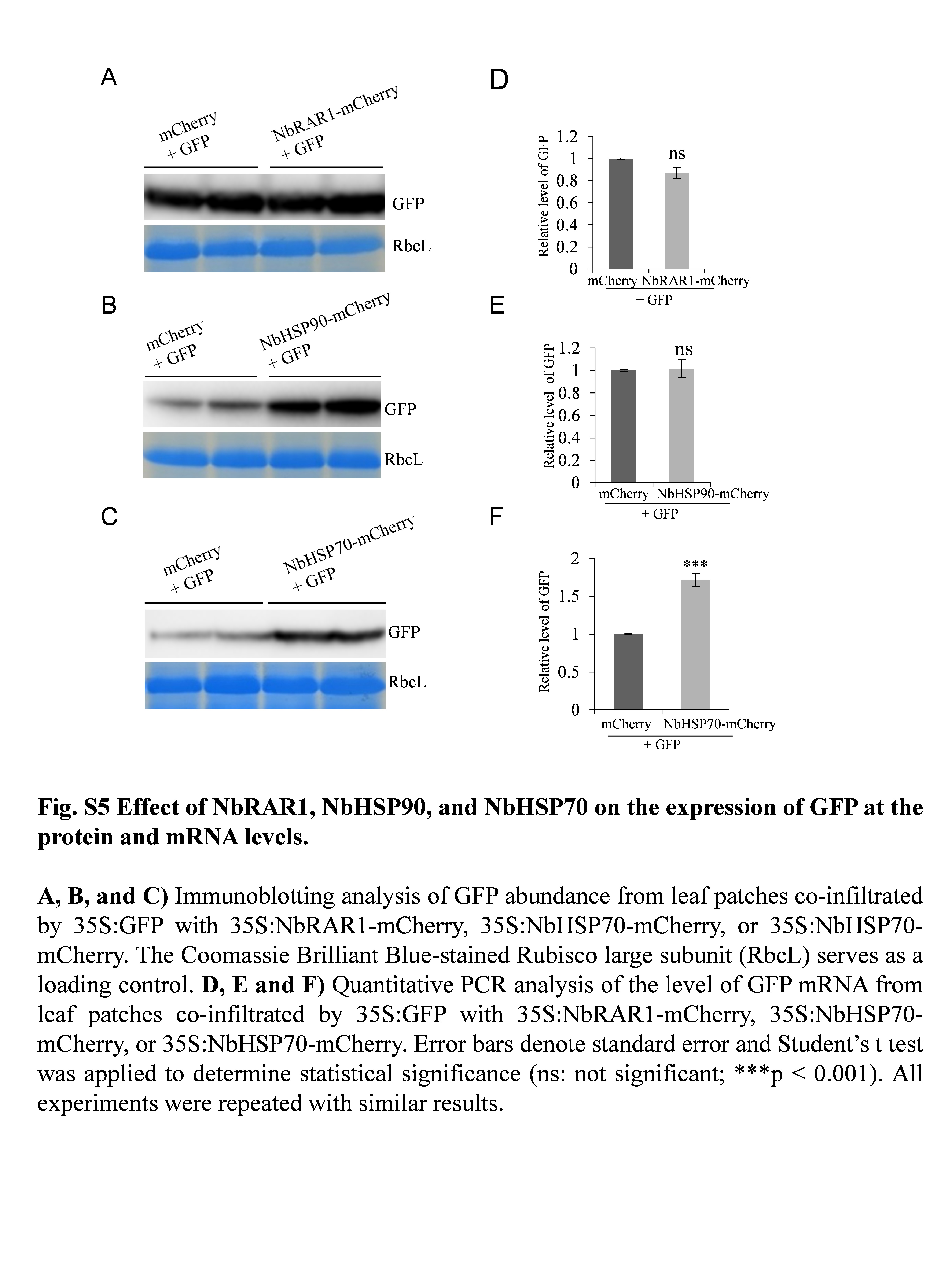

Supplement: Supplementary file 5 — Figure S5: Representative images showing the systemic infection of WT or mutant TelMV in N. benthamiana plants at 6 and 9 dpi. [file MPP-27-e70221-s007.jpg]

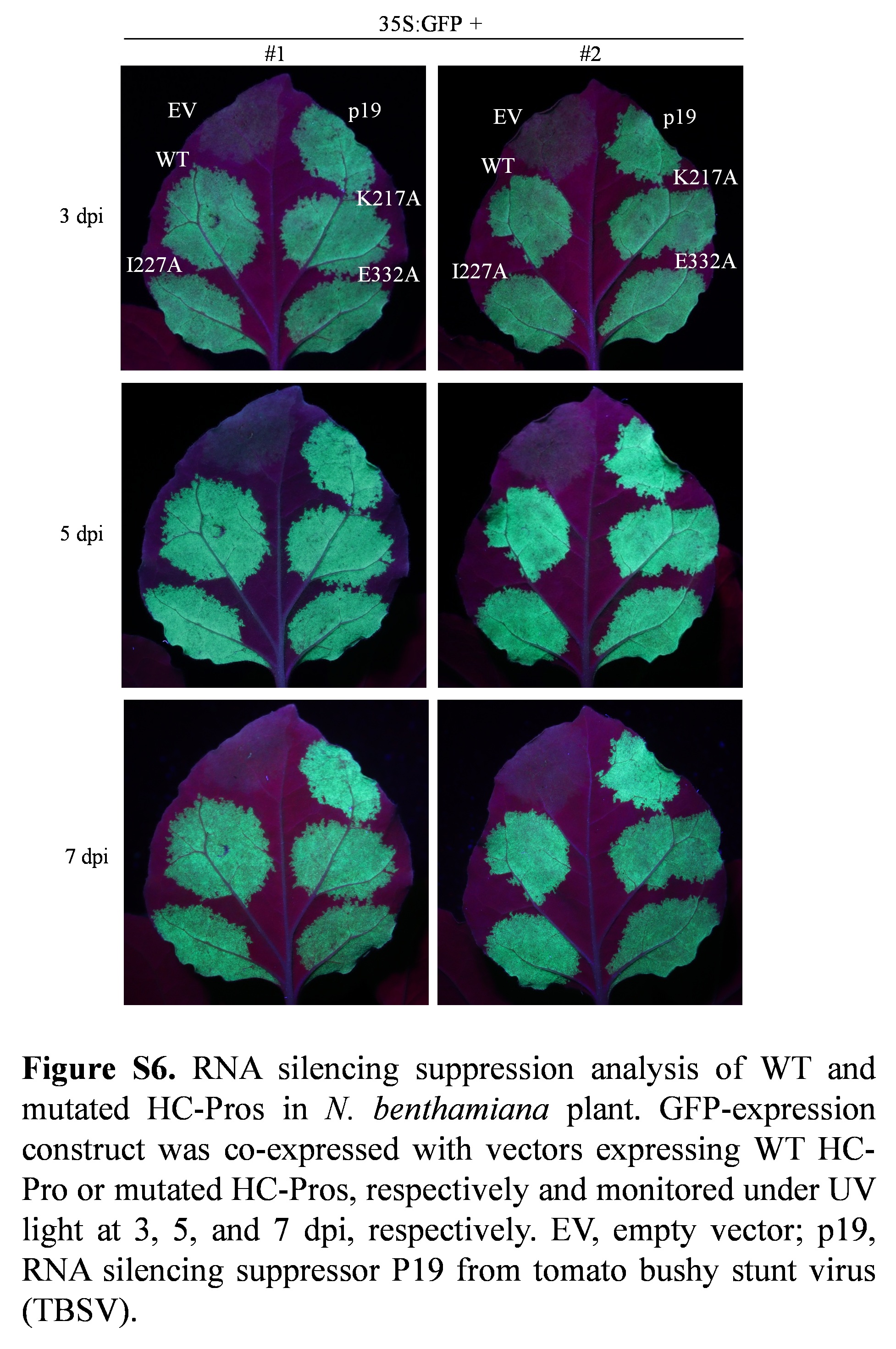

Supplement: Supplementary file 6 — Figure S6: RNA silencing suppression analysis of WT and mutated HC‐Pros in N. benthamiana plant. GFP‐expression construct was co‐expressed with vectors expressing WT HC‐Pro or mutated HC‐Pros, respectively and monitored under UV light at 3, 5, and 7 dpi, respectively. EV, empty vector; p19, RNA silencing suppressor P19 from tomato bushy stunt virus (TBSV). [file MPP-27-e70221-s006.jpg]

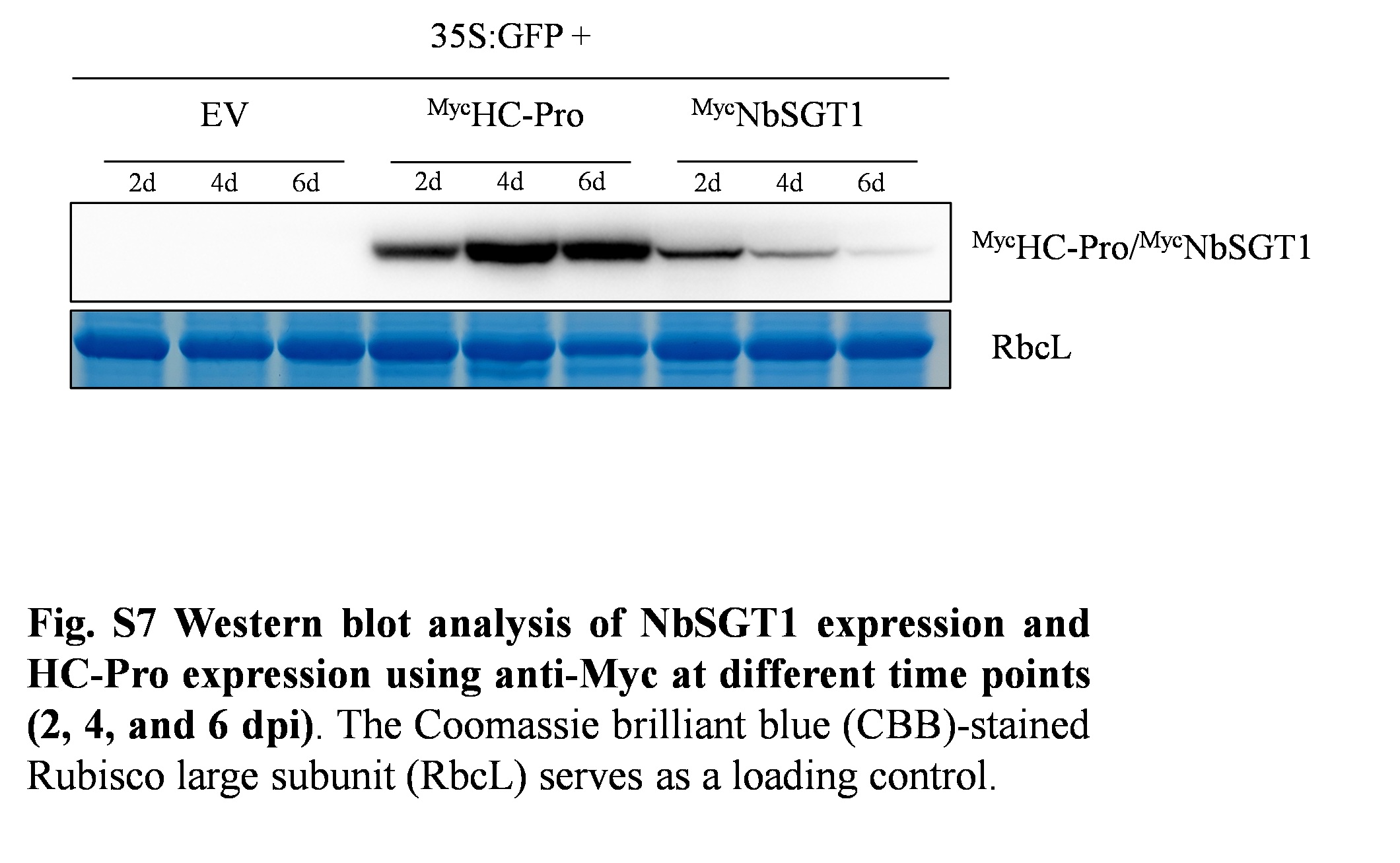

Supplement: Supplementary file 8 — Figure S8: Western blot analysis of NbSGT1 expression and HC‐Pro expression using anti‐Myc at different time points (2, 4, and 6 dpi). The Coomassie brilliant blue (CBB)‐stained Rubisco large subunit (RbcL) serves as a loading control. [file MPP-27-e70221-s003.jpg]
